# Supplementary figures and images for: Reduced coenzyme Q synthesis confers non-target site resistance to the herbicide thaxtomin A
Source: PLoS Genet. 2023 Jan 6;19(1):e1010423. doi: 10.1371/journal.pgen.1010423 (PMC9851558; doi:10.1371/journal.pgen.1010423)

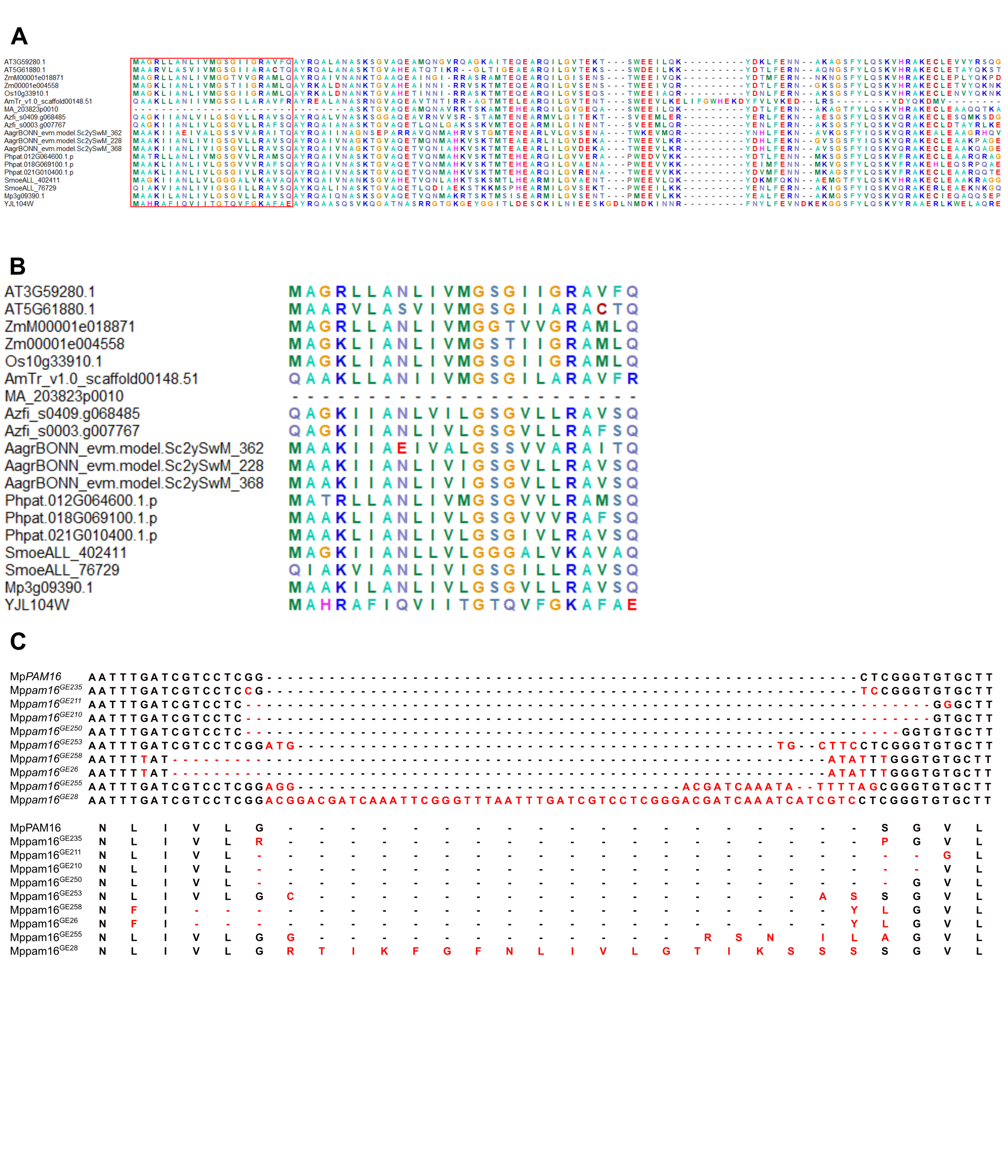

Supplement: S1 Fig — (A) Trimmed amino acid alignment of PAM16 homologues. Protein sequences similar to AtPAM16 (At3G59280) from 11 species were identified using the BlastP algorithm to search their proteomes [37]. The sequences were aligned via the L-INS-i strategy in MAFFT [76] and manually trimmed. The predicted signal peptides are marked in red and magnified in (B). (C) Mppam16 mutants were generated via CRISPR-Cas9 mutagenesis to target the MpPAM16 gene. Wild type spores (Tak-1 x Tak-2) were transformed with Agrobacterium tumefaciens strains carrying the vector containing the Cas9 gene and an sgRNA targeting MpPAM16. Positive transformants were genotyped using Sanger sequencing to determine the mutations induced by the CRISPR-Cas9 complex. Predicted protein sequences were determined based on the mutations in each line. Nucleotide or protein sequences were aligned via the L-INS-i strategy using MAFFT version 7. The top row is the reference MpPAM16 nucleotide sequence or the reference MpPAM16 protein sequence (MpTak v6.1). (TIF) [file pgen.1010423.s001.tif]

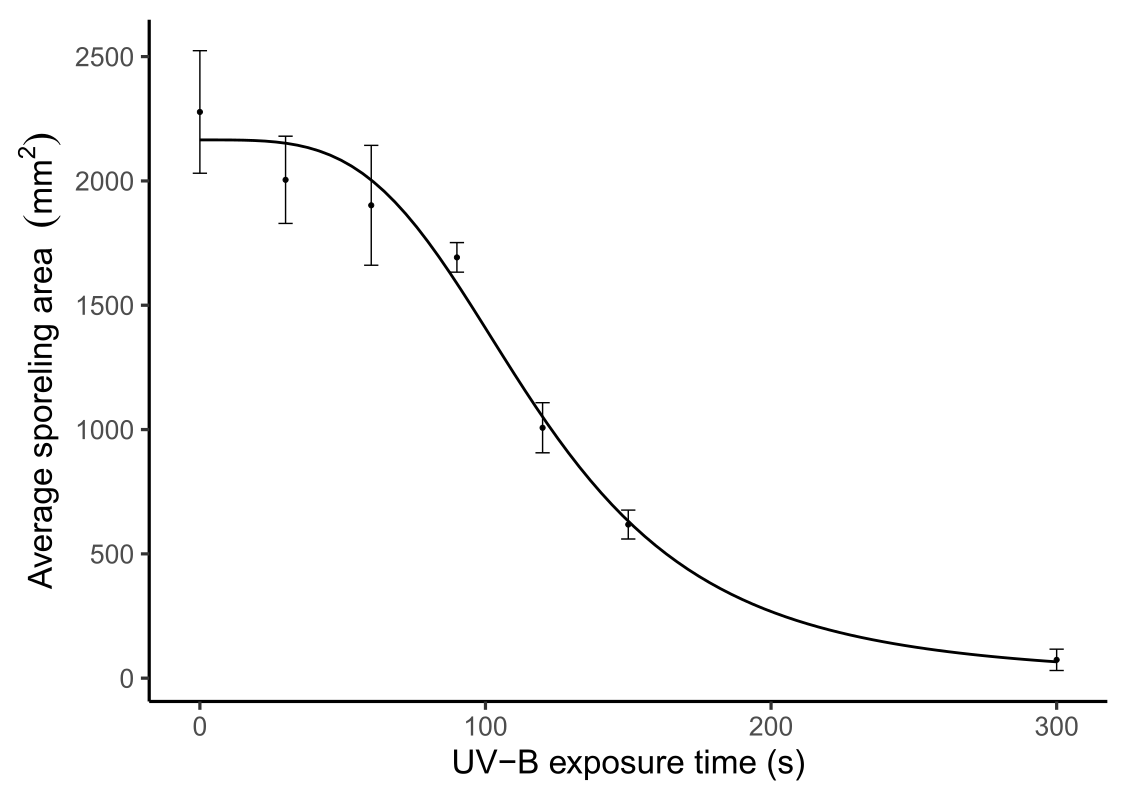

Supplement: S2 Fig — Wild type spores (from a cross between Tak-1 and Tak-2) were plated on solid medium and subjected to UV-B irradiation for different lengths of time using a UVP BioDoc-It. Spores were imaged after 14 days of growth using a Leica DFC310 FX stereomicroscope. The total area of spores on each plate was determined using ImageJ (Tab D in S1 Table). The fitted curve was calculated using the four-parameter log-logistic equation from the drc package in R. IC50 (exposure time of UV-B at which the average sporeling area on the plate was reduced by 50%) was calculated from the dose-response curve as approximately 110 s. Error bars represent ± standard deviation (n = 3). (TIF) [file pgen.1010423.s002.tif]

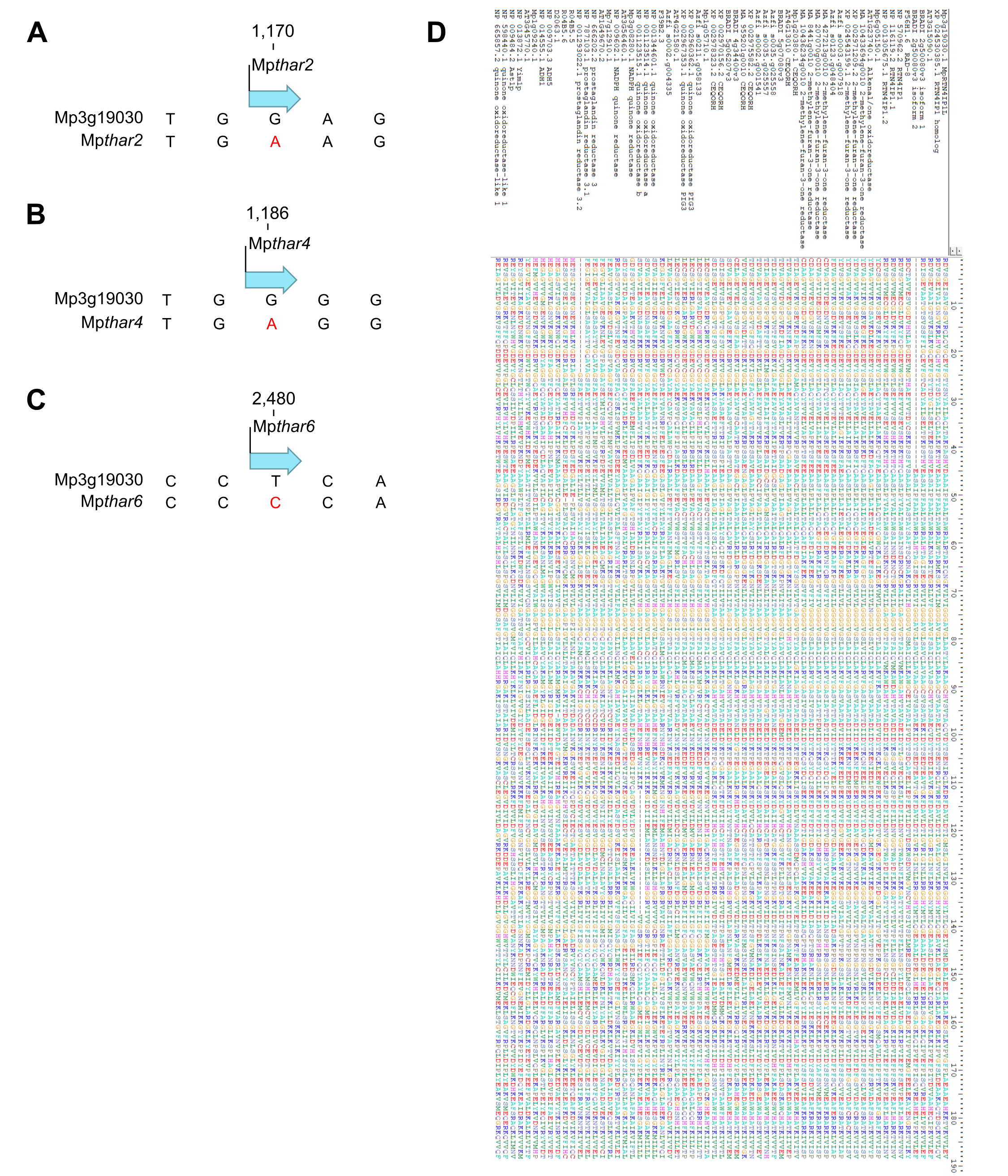

Supplement: S3 Fig — (A, B, and C) Regions of the Mp3g19030.1 gene in Mpthar2 (A), Mpthar4 (B), and Mpthar6 (C) were Sanger sequenced to confirm the SNPs identified by next generation sequencing. The consensus sequence is shown on the top rows, and results from the Sanger sequencing are shown on the second rows. The blue arrows indicate the site of mutation in each Mpthar mutant, and the tick marks above the sequences indicate the position of the nucleotide in the Mp3g19030.1 gene. (D) Trimmed amino acid alignment of RTN4IP1 homologues. Protein sequences similar to HsRTN4IP1 from 10 species were identified using the BlastP algorithm to search their proteomes [37]. The sequences were aligned via the L-INS-i strategy in MAFFT [76] and manually trimmed. (TIF) [file pgen.1010423.s003.tif]

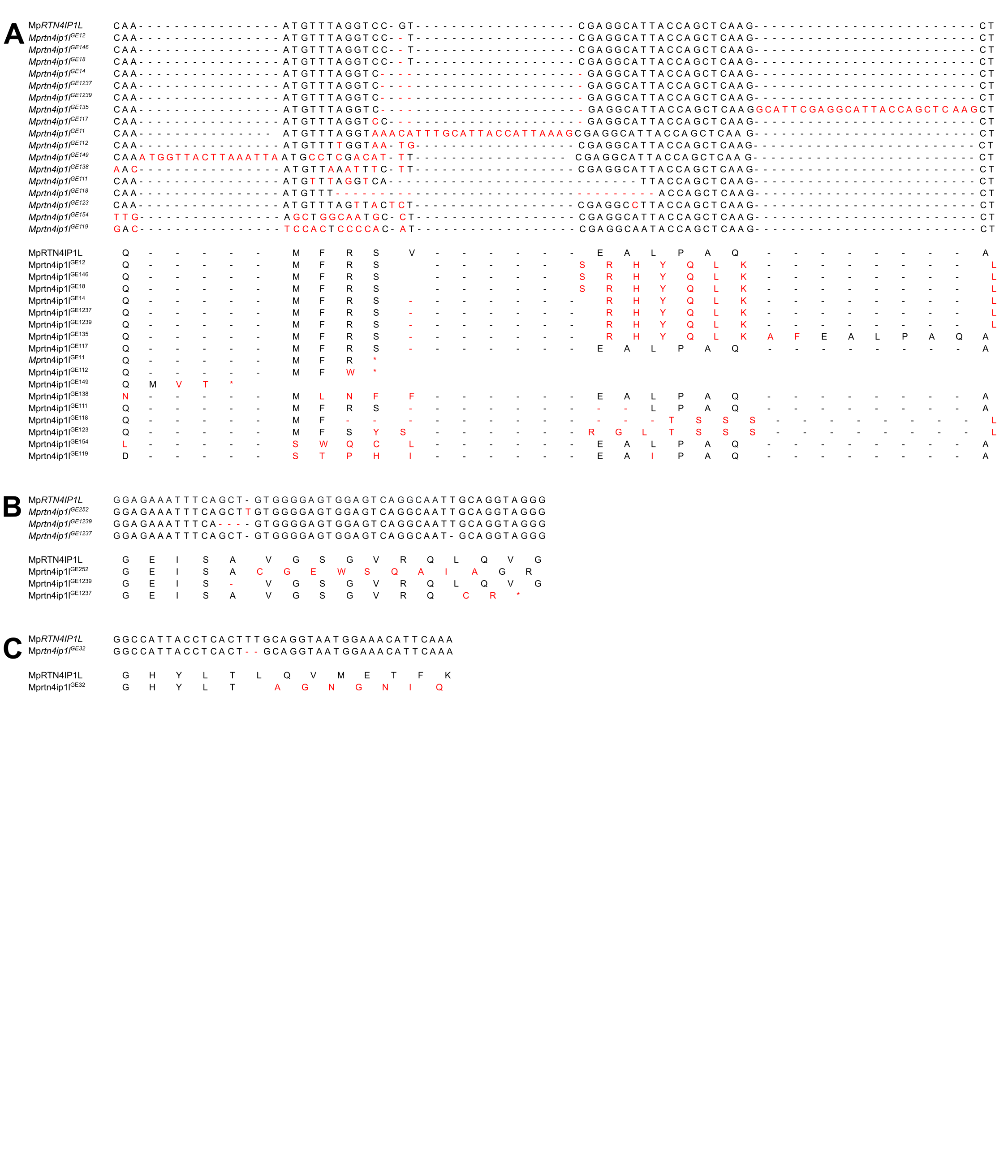

Supplement: S4 Fig — Mprtn4ip1l mutants were generated via CRISPR-Cas9 mutagenesis to target the MpRTN4IP1L gene. Wild type spores (Tak-1 x Tak-2) were transformed with Agrobacterium tumefaciens strains carrying the vectors containing the Cas9 gene and an sgRNA targeting MpRTN4IP1L: (A) sgRNA1, (B) sgRNA 2, (C) sgRNA3. Positive transformants were genotyped using Sanger sequencing to determine the mutations induced by the CRISPR-Cas9 complex. Predicted protein sequences were determined based on the mutations in each line. Nucleotide or protein sequences were aligned via the L-INS-i strategy using MAFFT version 7 [76]. The top row is the reference MpRTN4IP1L nucleotide sequence or the reference MpRTN4IP1L protein sequence (MpTak v6.1). (TIF) [file pgen.1010423.s004.tif]

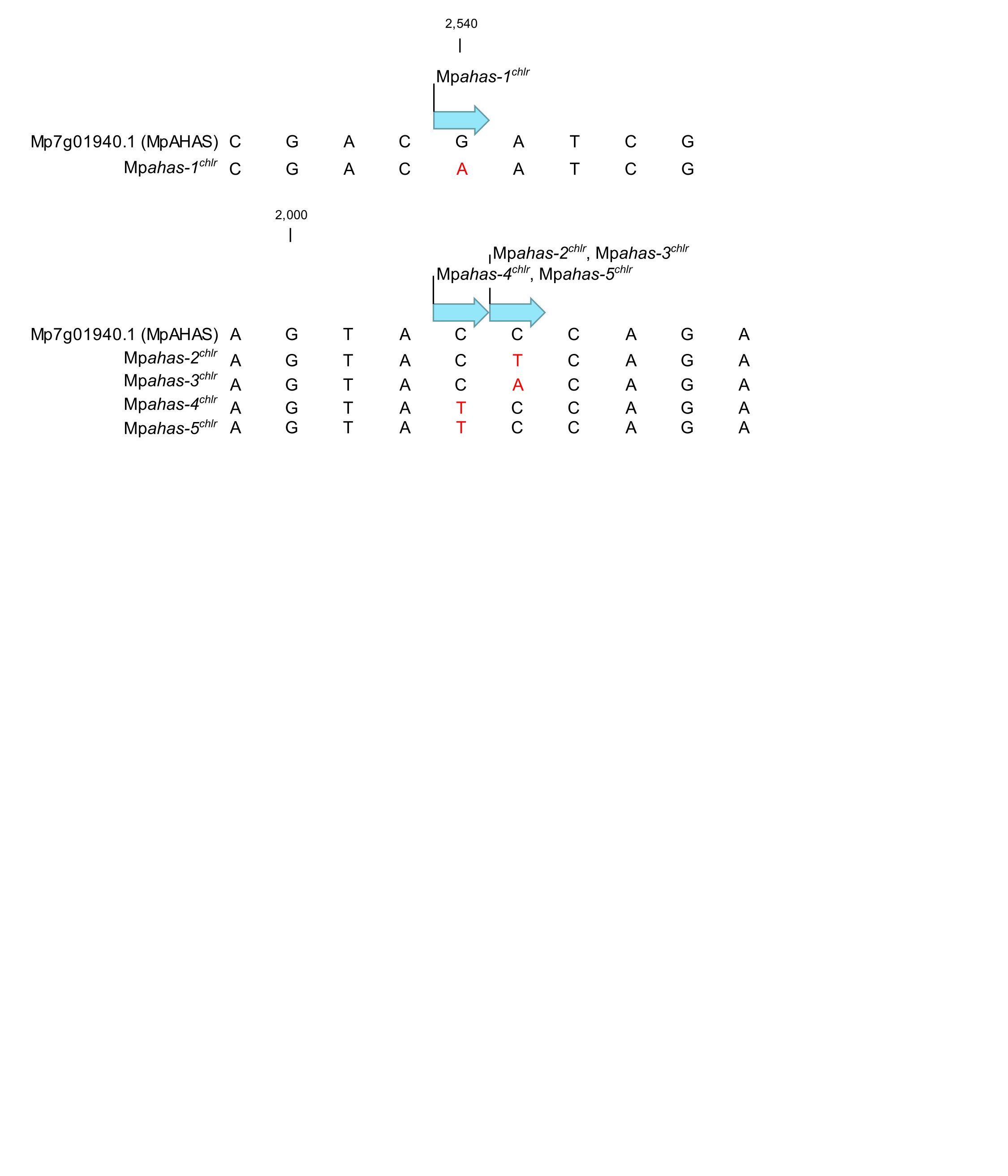

Supplement: S5 Fig — Regions of the MpAHAS gene (Mp7g01940.1) in each Mpahaschlr mutant were Sanger sequenced to identify any target-site resistance conferring mutations. The consensus sequence is shown on the top rows, and results from the Sanger sequencing are shown on the subsequent rows. The blue arrows indicate the site of mutation in each Mpahaschlr mutant, and the tick marks above the sequences indicate the position of the nucleotide in the MpAHAS gene. (TIF) [file pgen.1010423.s005.tif]

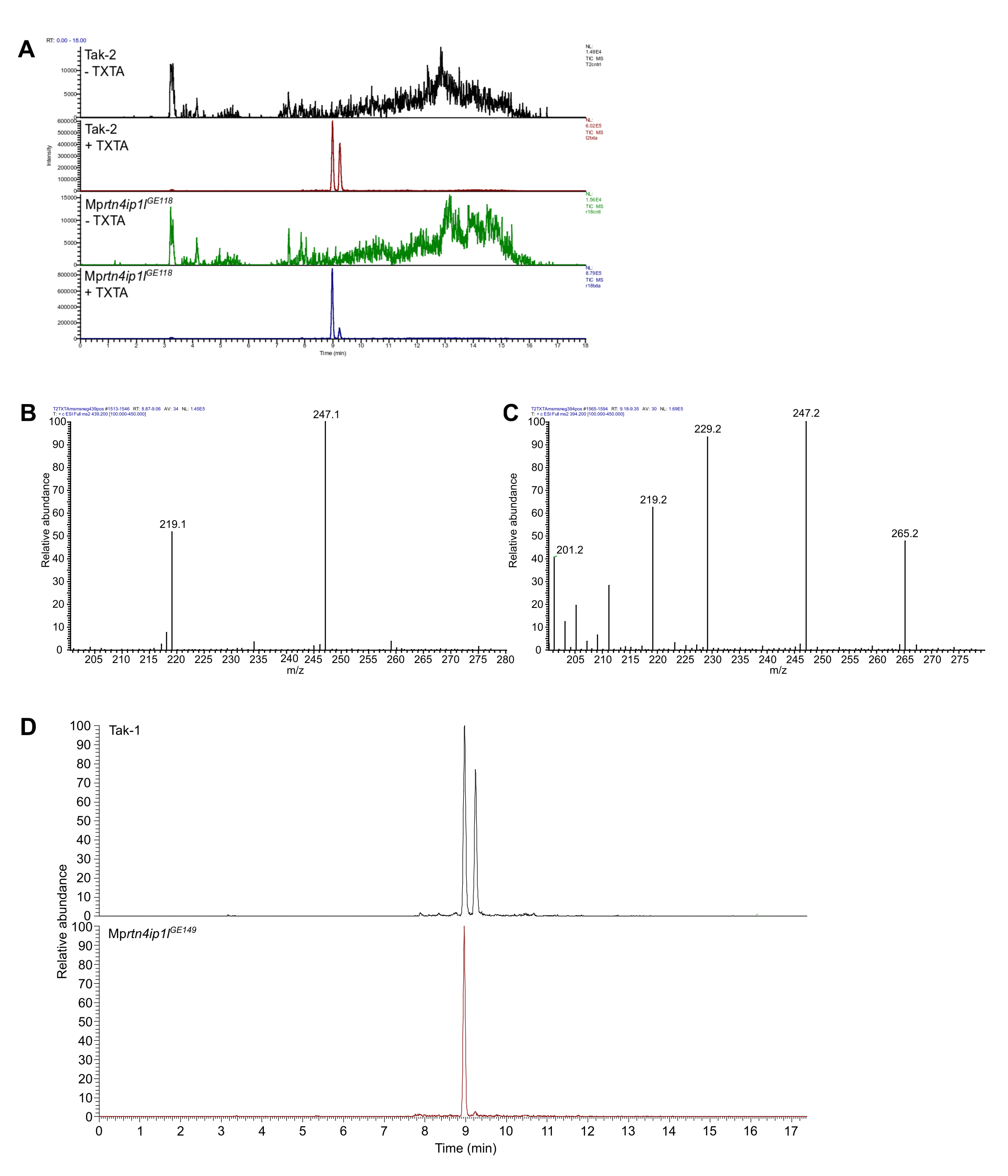

Supplement: S6 Fig — Gemmalings from each line were grown on solid medium supplemented with 0.1% DMSO for 14 days, then transferred to solid ½ Gamborg medium supplemented with 5 μM thaxtomin A or 0.1% DMSO and grown for 2 days. Cellular fractions were extracted from thaxtomin A-treated and untreated samples. (A) A precursor ion analysis of samples from Tak-2 and Mprtn4ip1lGE118 mutants was conducted via LC-MS/MS (n = 6). Chromatograms of the total ion currents of precursor ion scanning for m/z 247.1 are depicted. The peak eluting at a retention time of 8.99 min is Thaxtomin A (m/z 439.1), the peak eluting at a retention time of 9.25 min (m/z 394.1) is its putative metabolite. Both peaks are absent in the analysis of the untreated samples. (B) MS/MS of m/z 439.2 from thaxtomin A treated samples. The fragment ions m/z 219.1 and m/z 247.1 are identical to the fragment ions observed in the authentic standard of thaxtomin A and reported in the literature [83]. (C) MS/MS spectra of the unknown compound eluting at a RT of 9.25 min. Both prominent fragment ions of thaxtomin A are present. (D) A precursor ion analysis of samples from Tak-1 and Mprtn4ip1lGE149 mutants was conducted via LC-MS/MS (n = 6). Chromatograms of the total ion currents of precursor ion scanning for m/z 247.1 are depicted. The peak eluting at a retention time of 8.99 min is Thaxtomin A (m/z 439.1), the peak eluting at a retention time of 9.25 min (m/z 394.1) is its putative metabolite. (TIF) [file pgen.1010423.s006.tif]
