# Supplementary material for: Reduced coenzyme Q synthesis confers non-target site resistance to the herbicide thaxtomin A
Source: PLoS Genet. 2023 Jan 6;19(1):e1010423. doi: 10.1371/journal.pgen.1010423 (PMC9851558; doi:10.1371/journal.pgen.1010423)
Supplement: S1 Text — (RTF) [file pgen.1010423.s010.rtf]

##Fig1C_fastaseqs##>Mp3g09390.1MAAKILANLIVLGSGVLLRAVSQAYRQAIQNASKSGVAQETVQNMAKRTSKTMSISEARMILGVSENTPMEEVLKKYEALFERNAKMGSFYVQSKVQRAKECIEQAQQSEPQEQEHPTG>AT3G59280.1MAGRLLANLIVMGSGIIGRAVFQAYRQALANASKSGVAQEAMQNGVRQAGKAITEQEARQILGVTEKTSWEEILQKYDKLFENNAKAGSFYLQSKVHRAKECLEVVYRSQGNGTPS>AT5G61880.1 MAARVLASVIVMGSGIIARACTQAYRQALANASKTGVAHEATQTIKRGLTIGEAEARQILGVTEKSSWDEILKKYDTLFERNAQNGSFYLQSKVHRAKECLETAYQKSTTTSA>Phpat.012G064600.1.pMATRLLANLIVMGSGVVLRAMSQAYRQAIVNASKTGVAQETVQNMAHKVSKTMTEHEARQILGVVERAPWEDVVKKYDTLFENNMKSGSFYLQSKVFRAKECLEAARQRAGGQAQ>Phpat.021G010400.1.pMAAKLIANLIVLGSGIVLRAVSQAYRQAIVNASKTGVAQETVQNMAHKVSKTMTEHEARQILGVRENATWEEVVKKYDVMFENNMKAGSFYLQSKVFRAKECLEAARQQTEGQEQGQ>Phpat.018G069100.1.pMAAKLIANLIVLGSGVVVRAFSQAYRQAIVNASKTGVAQETVQNMAHKVSKTMTEHEARQILGVAENAPWEEVVKKYEIMFENNMKVGSFYLQSKVFRAKEHLEQSRPQAEGQAQEQGPGQ>SmoeALL_402411MAGKIIANLLVLGGGALVKAVAQAYKQAIVNANKSGVAHETVQNLAHKTSKSMTLHEARMILGVAENTPWEEVLQKYDKMFQKNAEMGTFYLQSKVHRAKECLEAAKRAGG>SmoeALL_76729MVGQVRVDQSGRENAAATNRSCFQIAKVIANLIVIGSGILLRAVSQAYKQALINASKTGVAQETLQDIAEKSTKKMSPHEARMILGVSEKTPWEEILKKYENLFERNAKIGSFYIQSKVQRAKERLEAEKNKGQ>AmTr_v1.0_scaffold00148.51MEEYWQAAKLLANIIVMGSGILARAVFRAYREALANASRNGVAQEAVTNTIRRAGTMTELEARQILGVTENTSWEEVLKELIFGWHEKDYFVLVKEDLRSVDYQKDMV>Os10g33910.1MAGKLIANLIVMGSGIIGRAMLQAYRKALDNANKTGVAHETINNIRRASKTMTEQEARQILGVSEQSTWEEIAQRYDNLFERNAKSGSFYLQSKVHRAKECLENVYQKNKQDGTPP>Zm00001e004558MAGKLIANLIVMGSTIIGRAMLQAYRKALDNANKTGVAHEAINNIRRVSKTMTEQEARQILGVSENSTWEEIVQRYDTLFERNGKSGSFYLQSKVHRAKECLETVYQKNKQDEPPN>ZmM00001e018871MAGRLLANLIVMGGTVVGRAMLQAYRQAIVNANKTGAAQEAINGIRRASRAMTEQEARQILGISENSTWEEIVQKYDTMFERNNKNGSFYLQSKVHRAKECLEPLYQKPDVLN>YJL104WMAHRAFIQVIITGTQVFGKAFAEAYRQAASQSVKQGATNASRRGTGKGEYGGITLDESCKILNIEESKGDLNMDKINNRFNYLFEVNDKEKGGSFYLQSKVYRAAERLKWELAQREKNAKAKAGDASTAKPPPNSTNSSGADNSASSNQ>Azfi_s0003.g007767MQAGKIIANLIVLGSGVLLRAFSQAYRQAIANASKTGVAQETLQNLGAKSSKVMTEQEARMILGINENTSVEEMLQRYENLFERNAKSGSFYLQSKVQRAKECLDTAYRLKESQKT>Azfi_s0409.g068485MAALVPLQAGKIIANLVILGSGVLLRAVSQAYRQALVNASKSGGAQEAVRNVVSRSTAMTENEARMVLGITEKTSVEEMLERYERLFEKNAESGSFYLQSKVQRAKECLESQMKSDGANGV>MA_203823p0010ASKTGVAQEAMQNAVRKTSKAMTEHEARQILGVGEQASWEEILQKYDTLFERNAKAGTFYLQSKVHRAKECLEAAQQTKACNGG>AagrBONN_evm.model.Sc2ySwM_368.3318.1MAAKIIANLIVIGSGVLLRAVSQAYRQAIVNAGKTGVAQETVQNIAHKVSKTMTEAEARLILGVDEKATWEEVLKRYDHLFERNAVSGSFYIQSKVQRAKECLEAAKQAGERATG>AagrBONN_evm.model.Sc2ySwM_228.728.1MAAKIIANLIVIGSGVLLRAVSQAYRQAIVNAGKTGVAQETMQNMAHKVSKTMTEAEARLILGVDEKATWEEVLKRYDHLFEKNGVSGSFYIQSKVQRAKECLEAAKPAGERATG>AagrBONN_evm.model.Sc2ySwM_362.2602.1MAAKIIAEIVALGSSVVARAITQAYRQAIINAGNSEPARRAVQNMAHRVSTGMTENEARLVLGVSENATWKEVMQRYNHLFEKNAVKGSFYIQSKVQRAKEALEAAGRHQVETR##Fig1C_aligned##>Mp3g09390.1-----------------------MAAKILANLIVLGSGVLLRAVSQAYRQAIQNASKSGVAQETVQNMAKRTSKTMSISEARMILGVSENT---PMEEVLKK---------YEALFERN--AKMGSFYVQSKVQRAKECIEQAQQSEPQEQEHP-TG------------------------->Phpat.012G064600.1.p-----------------------MATRLLANLIVMGSGVVLRAMSQAYRQAIVNASKTGVAQETVQNMAHKVSKTMTEHEARQILGVVERA---PWEDVVKK---------YDTLFENN--MKSGSFYLQSKVFRAKECLEAARQRAGGQAQ------------------------------>Phpat.021G010400.1.p-----------------------MAAKLIANLIVLGSGIVLRAVSQAYRQAIVNASKTGVAQETVQNMAHKVSKTMTEHEARQILGVRENA---TWEEVVKK---------YDVMFENN--MKAGSFYLQSKVFRAKECLEAARQQTEGQEQGQ---------------------------->Phpat.018G069100.1.p-----------------------MAAKLIANLIVLGSGVVVRAFSQAYRQAIVNASKTGVAQETVQNMAHKVSKTMTEHEARQILGVAENA---PWEEVVKK---------YEIMFENN--MKVGSFYLQSKVFRAKEHLEQSRPQAEGQAQEQGPG------------------------Q>AagrBONN_evm.model.Sc2ySwM_368.3318.1-----------------------MAAKIIANLIVIGSGVLLRAVSQAYRQAIVNAGKTGVAQETVQNIAHKVSKTMTEAEARLILGVDEKA---TWEEVLKR---------YDHLFERN--AVSGSFYIQSKVQRAKECLEAAKQAGERATG------------------------------>AagrBONN_evm.model.Sc2ySwM_228.728.1-----------------------MAAKIIANLIVIGSGVLLRAVSQAYRQAIVNAGKTGVAQETMQNMAHKVSKTMTEAEARLILGVDEKA---TWEEVLKR---------YDHLFEKN--GVSGSFYIQSKVQRAKECLEAAKPAGERATG------------------------------>SmoeALL_402411-----------------------MAGKIIANLLVLGGGALVKAVAQAYKQAIVNANKSGVAHETVQNLAHKTSKSMTLHEARMILGVAENT---PWEEVLQK---------YDKMFQKN--AEMGTFYLQSKVHRAKECLEAAKRAGG---------------------------------->SmoeALL_76729MVGQVRVDQSGRENAAATNRSCFQIAKVIANLIVIGSGILLRAVSQAYKQALINASKTGVAQETLQDIAEKSTKKMSPHEARMILGVSEKT---PWEEILKK---------YENLFERN--AKIGSFYIQSKVQRAKERLEAEKNKGQ---------------------------------->Azfi_s0003.g007767----------------------MQAGKIIANLIVLGSGVLLRAFSQAYRQAIANASKTGVAQETLQNLGAKSSKVMTEQEARMILGINENT---SVEEMLQR---------YENLFERN--AKSGSFYLQSKVQRAKECLDTAYRLKESQKT------------------------------>Azfi_s0409.g068485MAALVP----------------LQAGKIIANLVILGSGVLLRAVSQAYRQALVNASKSGGAQEAVRNVVSR-STAMTENEARMVLGITEKT---SVEEMLER---------YERLFEKN--AESGSFYLQSKVQRAKECLESQMKSDGANGV------------------------------>AT3G59280.1-----------------------MAGRLLANLIVMGSGIIGRAVFQAYRQALANASKSGVAQEAMQNGVRQAGKAITEQEARQILGVTEKT---SWEEILQK---------YDKLFENN--AKAGSFYLQSKVHRAKECLEVVYRSQGNGTPS----------------------------->MA_203823p0010------------------------------------------------------ASKTGVAQEAMQNAVRKTSKAMTEHEARQILGVGEQA---SWEEILQK---------YDTLFERN--AKAGTFYLQSKVHRAKECLEAAQQTKACNGG------------------------------>Os10g33910.1-----------------------MAGKLIANLIVMGSGIIGRAMLQAYRKALDNANKTGVAHETINNI-RRASKTMTEQEARQILGVSEQS---TWEEIAQR---------YDNLFERN--AKSGSFYLQSKVHRAKECLENVYQKNKQDGTPP---------------------------->Zm00001e004558-----------------------MAGKLIANLIVMGSTIIGRAMLQAYRKALDNANKTGVAHEAINNI-RRVSKTMTEQEARQILGVSENS---TWEEIVQR---------YDTLFERN--GKSGSFYLQSKVHRAKECLETVYQKNKQDEPPN---------------------------->ZmM00001e018871-----------------------MAGRLLANLIVMGGTVVGRAMLQAYRQAIVNANKTGAAQEAINGI-RRASRAMTEQEARQILGISENS---TWEEIVQK---------YDTMFERN--NKNGSFYLQSKVHRAKECLEPLYQKPD-------------------------------VLN>AT5G61880.1 -----------------------MAARVLASVIVMGSGIIARACTQAYRQALANASKTGVAHEATQTIKR--GLTIGEAEARQILGVTEKS---SWDEILKK---------YDTLFERN--AQNGSFYLQSKVHRAKECLETAYQKSTTTSA------------------------------>AagrBONN_evm.model.Sc2ySwM_362.2602.1-----------------------MAAKIIAEIVALGSSVVARAITQAYRQAIINAGNSEPARRAVQNMAHRVSTGMTENEARLVLGVSENA---TWKEVMQR---------YNHLFEKN--AVKGSFYIQSKVQRAKEALEAAGRHQVETR------------------------------->AagrBONN_evm.model.Sc2ySwM_362.2602.2-----------------------MAAKIIAEIVALGSSVVARAITQAYRQAIINAGNSEPARRAVQNMAHRVSTGMTENEARLVLGVSENA---TWKEVMQV---------GLHL-----------------------------EH------------------------------------>AmTr_v1.0_scaffold00148.51M------------------EEYWQAAKLLANIIVMGSGILARAVFRAYREALANASRNGVAQEAVTNTIRR-AGTMTELEARQILGVTENT---SWEEVLKELIFGWHEKDYFVLVKED--LRS--------VDYQKDMV------------------------------------------>YJL104W-----------------------MAHRAFIQVIITGTQVFGKAFAEAYRQAASQSVKQGATNASRRGTGKGEYGGITLDESCKILNIEESKGDLNMDKINNR---------FNYLFEVNDKEKGGSFYLQSKVYRAAERLKWELAQREKNAKAK-AGDASTAKPPPNSTNSSGADNSASSNQ##Fig1C_aligned_trimmed##>AT3G59280.1MAGRLLANLIVMGSGIIGRAVFQAYRQALANASKSGVAQEAMQNGVRQAGKAITEQEARQILGVTEKT---SWEEILQK---------YDKLFENN--AKAGSFYLQSKVHRAKECLEVVYRSQG>AT5G61880.1MAARVLASVIVMGSGIIARACTQAYRQALANASKTGVAHEATQTIKR--GLTIGEAEARQILGVTEKS---SWDEILKK---------YDTLFERN--AQNGSFYLQSKVHRAKECLETAYQKST>ZmM00001e018871MAGRLLANLIVMGGTVVGRAMLQAYRQAIVNANKTGAAQEAINGI-RRASRAMTEQEARQILGISENS---TWEEIVQK---------YDTMFERN--NKNGSFYLQSKVHRAKECLEPLYQKPD>Zm00001e004558MAGKLIANLIVMGSTIIGRAMLQAYRKALDNANKTGVAHEAINNI-RRVSKTMTEQEARQILGVSENS---TWEEIVQR---------YDTLFERN--GKSGSFYLQSKVHRAKECLETVYQKNK>Os10g33910.1MAGKLIANLIVMGSGIIGRAMLQAYRKALDNANKTGVAHETINNI-RRASKTMTEQEARQILGVSEQS---TWEEIAQR---------YDNLFERN--AKSGSFYLQSKVHRAKECLENVYQKNK>AmTr_v1.0_scaffold00148.51QAAKLLANIIVMGSGILARAVFRAYREALANASRNGVAQEAVTNTIRR-AGTMTELEARQILGVTENT---SWEEVLKELIFGWHEKDYFVLVKED--LRS--------VDYQKDMV-------->MA_203823p0010-------------------------------ASKTGVAQEAMQNAVRKTSKAMTEHEARQILGVGEQA---SWEEILQK---------YDTLFERN--AKAGTFYLQSKVHRAKECLEAAQQTKA>Azfi_s0409.g068485QAGKIIANLVILGSGVLLRAVSQAYRQALVNASKSGGAQEAVRNVVSR-STAMTENEARMVLGITEKT---SVEEMLER---------YERLFEKN--AESGSFYLQSKVQRAKECLESQMKSDG>Azfi_s0003.g007767QAGKIIANLIVLGSGVLLRAFSQAYRQAIANASKTGVAQETLQNLGAKSSKVMTEQEARMILGINENT---SVEEMLQR---------YENLFERN--AKSGSFYLQSKVQRAKECLDTAYRLKE>AagrBONN_evm.model.Sc2ySwM_362.2602.1MAAKIIAEIVALGSSVVARAITQAYRQAIINAGNSEPARRAVQNMAHRVSTGMTENEARLVLGVSENA---TWKEVMQR---------YNHLFEKN--AVKGSFYIQSKVQRAKEALEAAGRHQV>AagrBONN_evm.model.Sc2ySwM_228.728.1MAAKIIANLIVIGSGVLLRAVSQAYRQAIVNAGKTGVAQETMQNMAHKVSKTMTEAEARLILGVDEKA---TWEEVLKR---------YDHLFEKN--GVSGSFYIQSKVQRAKECLEAAKPAGE>AagrBONN_evm.model.Sc2ySwM_368.3318.1MAAKIIANLIVIGSGVLLRAVSQAYRQAIVNAGKTGVAQETVQNIAHKVSKTMTEAEARLILGVDEKA---TWEEVLKR---------YDHLFERN--AVSGSFYIQSKVQRAKECLEAAKQAGE>Phpat.012G064600.1.pMATRLLANLIVMGSGVVLRAMSQAYRQAIVNASKTGVAQETVQNMAHKVSKTMTEHEARQILGVVERA---PWEDVVKK---------YDTLFENN--MKSGSFYLQSKVFRAKECLEAARQRAG>Phpat.018G069100.1.pMAAKLIANLIVLGSGVVVRAFSQAYRQAIVNASKTGVAQETVQNMAHKVSKTMTEHEARQILGVAENA---PWEEVVKK---------YEIMFENN--MKVGSFYLQSKVFRAKEHLEQSRPQAE>Phpat.021G010400.1.pMAAKLIANLIVLGSGIVLRAVSQAYRQAIVNASKTGVAQETVQNMAHKVSKTMTEHEARQILGVRENA---TWEEVVKK---------YDVMFENN--MKAGSFYLQSKVFRAKECLEAARQQTE>SmoeALL_402411MAGKIIANLLVLGGGALVKAVAQAYKQAIVNANKSGVAHETVQNLAHKTSKSMTLHEARMILGVAENT---PWEEVLQK---------YDKMFQKN--AEMGTFYLQSKVHRAKECLEAAKRAGG>SmoeALL_76729QIAKVIANLIVIGSGILLRAVSQAYKQALINASKTGVAQETLQDIAEKSTKKMSPHEARMILGVSEKT---PWEEILKK---------YENLFERN--AKIGSFYIQSKVQRAKERLEAEKNKGQ>Mp3g09390.1MAAKILANLIVLGSGVLLRAVSQAYRQAIQNASKSGVAQETVQNMAKRTSKTMSISEARMILGVSENT---PMEEVLKK---------YEALFERN--AKMGSFYVQSKVQRAKECIEQAQQSEP>YJL104WMAHRAFIQVIITGTQVFGKAFAEAYRQAASQSVKQGATNASRRGTGKGEYGGITLDESCKILNIEESKGDLNMDKINNR---------FNYLFEVNDKEKGGSFYLQSKVYRAAERLKWELAQRE##Fig1C_tree##(MA_203823p0010:0.09592970,(((AagrBONN_evm.model.Sc2ySwM_362.2602.1:0.46275084,(AagrBONN_evm.model.Sc2ySwM_368.3318.1:0.00923364,AagrBONN_evm.model.Sc2ySwM_228.728.1:0.03738023)0.718000:0.03161416)0.950000:0.09465700,((SmoeALL_402411:0.27121427,Mp3g09390.1:0.18087597)0.733000:0.02856259,(SmoeALL_76729:0.28864899,(Azfi_s0409.g068485:0.32026384,Af2:0.13453853)0.952000:0.15283083)0.610000:0.03765304)0.770000:0.02347661)0.892000:0.06691454,(Phpat.012G064600.1.p:0.10330108,(Phpat.021G010400.1.p:0.03876558,Phpat.018G069100.1.p:0.08817130)0.765000:0.02378271)0.796000:0.07709764)0.971000:0.18940005,(((AT3G59280.1:0.14408230,(AT5G61880.1:0.16034217,YJL104W:2.04176563)0.751000:0.12734614)0.851000:0.05284687,AmTr_v1.0_scaffold00148.51:0.63145848)0.000000:0.00000001,(ZmM00001e018871:0.20251012,(Os1:0.05830950,Zm00001e004558:0.02029987)0.556000:0.07235902)0.965000:0.13135792)0.900000:0.12175154);##Fig3C_fastaseqs##>Mp3g19030.1 MpRTN4IP1LMRRQSVQMFRSVEALPAQACIGSFRGLSTCTAVLVRRFGGPEVMEYRNGVALPDLGPNDVLVRARAVSVNPLDIRMRGGYGKSLFQPLLPIVLGRDVSGEISAVGSGVRQLQVGQEVFGALHPTAIRGTYSNYAILAEEQLTAKPASLSHVDAAAIPFAALTAWRALRSTARIQNGQKVLVIGGGGAVGLAATYLAKAAGCHVSVTCGKKSVERVMEAGAQQAVDYTSENIREQLKDRFDAVLDTIGMPETESLGINVLRKGGHYLTLQGEIVSMADKYGLLAGGAAAAAKLVQKQVQYKQSHGIEYWWTVMRTDAEALEEIARLAKEGSLKVPVGKTFPLAEAAEAHKAREGKKTVGKVVLEVE>XP_024530385.1 RTN4IP1 homologMRRWLASPYCRGHSRSISTCRAVVLPRFGGPEVLELRKDVPVPHLEAEEVLVRTVAAGVNPLDIKMREGYGRSLFQPLLPIVLGRDVSGEIVAIGNGVKRLHVGQHVFGALHPTAVRGTYSDYAILQEEQLALKPKSLTHVEAAAIPFAALTAWRALWSTARMKSGQKVLIMGGGGAVGLAAIQLARAAGCSVAATCGKRSCSRVGEAGAELIVDYTSENTREQLTWRFDAVLDTIGVETTEALGVNVLKRGGHYMTLQGEAVKLADKYGLIVGGGIATATLLRKRMQYTQSHGIDYSWIIMRTDAEGLEQIARLAKEGRLKIPVGETFSLDEAAKAQTARENKDSQGKVVLQVQQE>AT3G15090.1MRVMRSLRGNSGAGLVFRPARLNSLRSVFTGCRAVILPRFGGPEVFELRENVPVPNLNPNEVLVKAKAVSVNPLDCRIRAGYGRSVFQPHLPIIVGRDVSGEVAAIGTSVKSLKVGQEVFGALHPTALRGTYTDYGILSEDELTEKPSSISHVEASAIPFAALTAWRALKSNARITEGQRLLVFGGGGAVGFSAIQLAVASGCHVTASCVGQTKDRILAAGAEQAVDYTTEDIELAVKGKFDAVLDTIGGPETERIGINFLRKGGNYMTLQGEAASLTDKYGFVVGLPLATSLLMKKKIQYQYSHGIDYWWTYMRADPEGLAEIQRLVGAGKLKIPVEKTFPITDVVAAHEAKEKKQIPGKVVLEF>F56H1.6 RAD-8MIEKMILRRFFSTKSSTMRAWVSENGSGVELKEVPLPVINKPGQVLLKVKAASVNPIDVDMSQGYGREFLGTWKKIESCDAAASRFPLIPGRDCTAVVESVGGDVHNLAPGDEVMAVVPVILPGTHAEFVVTDSKYCSKKPSNLSFVSAAALPYVASTAYSAFTIARVSQRNAKQQRVLIHGGAGGVGSMAIQLLKAWGCEKIVATCAKGSFDIVKQLGAIPVDYTSDQATQELIEHAPFEVILDTVDSQLAKWSDNVMGVWRNCVHVSIVSPLMREMDKNGVPLGLVTTAMKHFERSFQSHLRGRWFSYAFFRPSSDLMSQLSRFAEDGKIVPVVEQVMGFEELEKAYEKVSQLNGRGKTVIKYD>NP_570962.2 RTN4IP1MGVLKTCVLRRSACAAACFWRRTVIPKPPFRGISTTSARSTVMPAWVIDKYGKNEVLRFTQNMMLPIIHYPNEVIIKVHAASVNPIDVNMRSGYGATALNMKRDPLHMKTKGEEFPLTLGRDVSGVVMECGLDVKYFQPGDEVWAAVPPWKQGTLSEFVVVSGNEVSHKPKSLTHTQAASLPYVALTAWSAINKVGGLSDRNCKGKRALILGASGGVGTFAIQVMKAWGAHVTAVCSKDASELVRKLGADEVIDYTLGSVEEQLKSLKLFDFILDNVGGSTETWALNFLKKWSGATYVTLVTPFLLNMDRLGVADGMLQTGVTVGTKALKHLWQGVHYRWAFFMASGPYLDEIAELVDAGKIRPVIERTFPFSEVPEAFLKVERGHARGKTVVNVV>NP_116119.2 RTN4IP1.1MEFLKTCVLRRNACTAVCFWRSKVVQKPSVRRISTTSPRSTVMPAWVIDKYGKNEVLRFTQNMMMPIIHYPNEVIVKVHAASVNPIDVNMRSGYGATALNMKRDPLHVKIKGEEFPLTLGRDVSGVVMECGLDVKYFKPGDEVWAAVPPWKQGTLSEFVVVSGNEVSHKPKSLTHTQAASLPYVALTAWSAINKVGGLNDKNCTGKRVLILGASGGVGTFAIQVMKAWDAHVTAVCSQDASELVRKLGADDVIDYKSGSVEEQLKSLKPFDFILDNVGGSTETWAPDFLKKWSGATYVTLVTPFLLNMDRLGIADGMLQTGVTVGSKALKHFWKGVHYRWAFFMASGPCLDDIAELVDAGKIRPVIEQTFPFSKVPEAFLKVERGHARGKTVINVV>NP_001305675.1 RTN4IP1.2MKRDPLHVKIKGEEFPLTLGRDVSGVVMECGLDVKYFKPGDEVWAAVPPWKQGTLSEFVVVSGNEVSHKPKSLTHTQAASLPYVALTAWSAINKVGGLNDKNCTGKRVLILGASGGVGTFAIQVMKAWDAHVTAVCSQDASELVRKLGADDVIDYKSGSVEEQLKSLKPFDFILDNVGGSTETWAPDFLKKWSGATYVTLVTPFLLNMDRLGIADGMLQTGVTVGSKALKHFWKGVHYRWAFFMASGPCLDDIAELVDAGKIRPVIEQTFPFSKVPEAFLKVERGHARGKTVINVV>Mp6g05150.1MAGVVTAGVGGNVAVLAGQCDGRQRRRRSSSSCKASMSLVPATKFGGAVGLRTKENGGRAWRRELNSSAVLGVRAEIRTEEASADSVAMAIPQTQQAWVYQAYGEKEVMSIGEVAVPELNPDQVLIKVCAAALNPVDSKRRGGKFQATDSDLPIVPGYDCSGIVVKVGSEVSKFKVGDEVYGDTSEFCLNGPKQYGTLAQYTAAEEKLLAKKPQSLTFAEAASLPLALQTAYQGWQKAGIKEGHKVMVLGGAGGVGTLAIQLAKQVFKASSVAATASTGKVDLLKSLGADVVVDYTKQKLEEWPEKYDVVYDTVGKGEGTKVVKPDGGLVVITGPVEPPGFRYVIVSKGSDLEELNPYFETGSMQAVLDPQGLFPFTKVVEAFGYLETGRAFGKVVISPIE>AT1G23740.1 Alkenal/one oxidoreductaseMNAALATTTATTPVLRRETPLLHYCSLTTKSPVYQINRVRFGSCVQTVSKKFLKISASSQSASAAVNVTADASIPKEMKAWVYSDYGGVDVLKLESNIVVPEIKEDQVLIKVVAAALNPVDAKRRQGKFKATDSPLPTVPGYDVAGVVVKVGSAVKDLKEGDEVYANVSEKALEGPKQFGSLAEYTAVEEKLLALKPKNIDFAQAAGLPLAIETADEGLVRTEFSAGKSILVLNGAGGVGSLVIQLAKHVYGASKVAATASTEKLELVRSLGADLAIDYTKENIEDLPDKYDVVFDAIGMCDKAVKVIKEGGKVVALTGAVTPPGFRFVVTSNGDVLKKLNPYIESGKVKPVVDPKGPFPFSRVADAFSYLETNHATGKVVVYPIP>MA_10433694g0010 2-methylene-furan-3-one reductaseMDTFAATTITARTCINSFPRAGGADDIQSHVKFGKIGFGWNQRGQSSYKLRHKSSNRTRGSRGVSVYAMADSAAAVDQKTEKFVVPSVQKAWIYKEYGAAKDVLQLDDGVPVPEVKEDQVLIKVCAAALNPVDFKRRQGKFKATDSPLPTVPGYDVAGIVIKVGSKVEGFKEGDEVYGDINEKALDNPKQYGSLGEYTAVEEKLVALKPKNLDFTQAASLPLAILTAYEGLERAGFGPGKSILVLGGAGGVGSLVIQLARKVFGASTIAATSSTGKLEFLKSLGTDISIDYTKQNFEELPEKYDVIYDAVGQCDKAVKAIKGGGSVVALTGAVTPPGFRFVVTSNGSVLSKLNPYLESGEIKPIIDPKGSFSFSQLVEAFSYLETCRATGKVVISPIP>XP_002971209.2 2-methylene-furan-3-one reductaseMHPDLAMESVCVSQSPWFPVGQLSRSKKRARATLTFASSSSKIGEMASTVAAIPSLHKAWVYKEYGAAKDVLKLEELPVPPLAPDQVLVKVHAAALNPVDFKRRLGKFKATDSDLPTVPGYDVAGVVAKVGSEVSSLKQGDEVYGDISEFALDKPKQWGSIAQYTAVEEKLLAIKPRNLSFIQAASLPLAIQTAQEGFDKAKLRAGQSVLVLGGAGGVGSLAIQLAKFVYGASLVAATTSTPKLELVKSLGADIVIDYKEKNFEDMPERYDVVFDAVGEPKGTNVVKEGGTAVVLTGPVNPPGFRFVVTSNGSNLVRLKDFLESGTVKPVIDPKGPFDFGSVVDAFCYLEGGRASGKVVISVLQ>XP_024543199.1 2-methylene-furan-3-one reductaseMHPDLAMESVCVSQSPWFPVGQLSRSKKRARATLTFASSSSKIGEMASTVAAIPSLHKAWVYKEYGAAKDVLKLEELPVPPLAPDQVLVKVHAAALNPVDFKRRLGKFKATDSDLPTVPGYDVAGVVAKVGSEVSSLKQGDEVYGDISEFALDKPKQWGSIAQYTAVEEKLLAIKPRNLSFIQAASLPLAIQTAQEGFDKAKLRAGQSVLVLGGAGGVGSLAIQLAKFVYGASLVAATTSTPKLGLVKSLGADIVIDYKEKNFEDMPERYDVVFDAVGEPKGTNVVKEGGTAVVLTGPVNPPGFRFVVTSNGSNLVRLKDFLESGTVKPVIDPKGPFDFGSVVDAFCYLEGGRACGKVVISVLQ>Azfi_s0003.g007918MEFVVAISHCPRSPLVQCCSGIELRTSSIANLNNRHNSFRASVNSHSHLINPTTSKTQLKKSSHYPPHGRIHAKMISETEVSSSVAEIPAVQKAWFYEEYGDDVLKFGEVPVPQILPDQVLIKVHAAALNAVDSKRRTGKFKATDSPLPTVPGYDVSGVIVKVGSEVTSFKEGDEVYANISEAALNGPKQWGSIAQYTVTEEKLLGIKPKNLSFAEAASLPLAIETALEGLERAGFKEGQTLLVLGGAGGVGSLAIQLAKQVFGASLVAATTSTPKVEFVKSLGADVVIDYKKEKFEDLPEKYDVVFDGVGECEKAVKVVKEGGSVIALTGPVTPPGFRFVVTSKGSSLAKLNPYLESGKVKPILDPKGQFSFSQVVEAFSYIGTGRATGKVVIAPIE>Azfi_s0123.g048404MGSLQKAWFYRDYGSVEVLEYGDVEVPQITSPDQVLIKVHAASLNPLDYKRRFGYIKSPTIPFPHVPGFDVAGVVVKVGSDVKKFKEGDEVYGDIAEFAIAAPKQIGTLAQYTVTEEKVLALKPKSLSFEEAASLPLALLTAQTGFEKAAFKPGQSVLILGGAGGVGTLAIQLAKHVYGASLVATTTSTGKLEFVKSLGADVAIDYTTTKYYELPEKYDFVYDTVGDGANAAKAVKEGGAVLTIADHSAKPPVIAYGLIANGDNLAKLNPFLEEGKLKPVIDPKGAFPFSQVKEAFEYLETSRARGKLVIAPIH>MA_427213g0010 2-methylene-furan-3-one reductaseMDYMQKAWFYKEYGTREVLQYGDFPVPQLGDNQVLVRVRAAALNPVDFKLRNGEIKDTGLEFPVVPGCDVAGVIVDEGDNVSKFSKGDEVYGNIQNFNTGRPKQCGTLTQYTAVEESLIASKPENMSFEEAASFPLALQTAQEGFDRVNFQRGQSVFIVGGAGGVGSLAIQLAKYVYGASKIATTTSTGKVDFVRQLGADTVIDYTKQSYDQIPQKFDFVFDTVGDSHRSYVVAKEKAKIIDIANPSPHPRAEFFIVTASGSNLERLRGYIECEKLKPVIDPISPYSFSNVMEAFKHLESGRARGKIVISPID>MA_207070g0010 2-methylene-furan-3-one reductaseMDYMQKAWFYNEYGPRAVLQYGDFPVPQLGDTQVLVRVRAAALNPVDFKLRNGVFKDTGLEFPVVPGCDVAGVIVDEGDNVSMFSKGDEVYGNIQSFNTVGPKQYGTLAQYTAVEESLIALKPENMSFEEAASFPLALQTAQEGFDRAKFLRGQTVFIVGGEGGVGSLAIQLAKYVYGASKIATTTSPGKVDFVKKLGPDMVIDYTKKSYDQILEKFDFVFDTIGDSHRSYVVAKEEGKIIDIANSSPHPRAEFYIVTPSGSNLERLRSYIECEKLKPVIDPTGPYAFSNVMEAFKHLESGRARGKVVISPID>MA_8441g0010 2-methylene-furan-3-one reductaseMSKLQKAWFYNEYGSIDVLQFGEVPVPTPGPGQLLVKIRAAALNPVDFKRRDGLFRNKDSDFPAVPGCDAAGVVVEVGDGVSKFKNGDEVYSDIQDFGAGRPKQWGTLAQYTVVEEYLVAPKPTNLSFEEAASLPLALLTAQQAFDIAKFEKGKSVFIVGGAGGVGSLAIQLARHVYGASKIVSTASTGKLDFVKSLGADLVIDYTKQSYDQISEKFDFVFDTIGDSAKSHVVAKEEAKILDIASFQPTSRVEFFGVSPHAKNLEKLQPYIESKKLKPVIDPKSPYSFSDVIEAFKHQESGRARGKIVISPIE>MA_10436234g0010 2-methylene-furan-3-one reductaseMSKLQKAWFYNEYGSIDVLQFGEVPVPTPGPGQLLVKIRAAALNPVDFKRRDGRIRIADSDFPTVPGCDAAGVVVDVGDGVSKFKNGDEVYSDIQNFVVGKRKQWGTLAQYTVVEEYLVAPKPTNLSFEEAASLPLALLTAQQAFDIAKFEKGKSVFIVGGAGGVGSLAIQLARHVYGASKIVSTASTGKLDFVKSLGADLVIDYTKQSYDQISEKFDFVFDTIGDSAKSHVVAKEEAKILDIASFQPTSRVEFFGVSPHAKNLEKLQPYIESKKLKPVIDPKSPYSFSDVIEAFKHQESGRARGKIVISPIE>Mp1g20380.1 CEQORHMAEMMQAVQYSSYGGAHAALQHKEIPIPVPKKGEILVKVEAASVNPVDWKMQGGVMRPMLPAKFPHTPGTDIAGEVVKVGSEVKDFAPGDKVVAWLELKHGGAMAQYAVANPKTTVVRPPEVSAVEAACLPVAALTALQALTSGGMNFDGSYSGNVLVTGASGGVGTYAVQLGKIAGAHVTATCGERNIELIKFLGADEVLDYKTPEGKKLISPSGKKYDLVVNAAASAVTFSDMQPQLAPKGVVFELTPSPKTFLTSVIKRVSMSKQKYNQLMLNVEARNLEMLVGFVKEGKLHAVIDSKFPLAKVEEAWKKSKEGHAVGKIVITVTEE>AT4G13010 CEQORHMAGKLMHALQYNSYGGGAAGLEHVQVPVPTPKSNEVCLKLEATSLNPVDWKIQKGMIRPFLPRKFPCIPATDVAGEVVEVGSGVKNFKAGDKVVAVLSHLGGGGLAEFAVATEKLTVKRPQEVGAAEAAALPVAGLTALQALTNPAGLKLDGTGKKANILVTAASGGVGHYAVQLAKLANAHVTATCGARNIEFVKSLGADEVLDYKTPEGAALKSPSGKKYDAVVHCANGIPFSVFEPNLSENGKVIDITPGPNAMWTYAVKKITMSKKQLVPLLLIPKAENLEFMVNLVKEGKVKTVIDSKHPLSKAEDAWAKSIDGHATGKIIVEP>Azfi_s0035.g025558MASSKIMQALQYSSYGGGASSLKHVEIPVPTPKSGEILVKIEAASVNPVDWKIQSGIARPFIPFKFPYVPGTDIAGEVVSVGPGVNTFSVGDKVISWLELNKGGAFAEFAVASLKYTAKIPEGVTAIDGCSLPVAAITALQAIRDANGAGIKLDGTSKANLLITAASGGVGIYAVQIAKLTGAHVTATCGARNIDLVKNLGANEVLDYKTPEGDALKSPSGVKYDAIIHCATGIPWSKFSSTLTPNGKVIDLTPNFKSIVHTIFKKVTFSKQKLVPFMASPNSEDLKLLGELVKDGKLRNIVDSQHPFAKAEESWVRSMEGHATGKVVVTFQE>Azfi_s0035.g025557MNVYVRKRDFAAQKDLTFRQVVRKIIYVSVISFLAGYAGTDIAGEVVSVGPGVKTFSVGDKIMSFLDLMKGGSLAEFAIAIPKFTVKRPEGVSAIDGCSLPVAGMSALQAIRNSNGAGIKLDGASKANLLITAASGGVGTYALQIAKLTGAHVTGTCGARNIELVKSLGADEILDYKTPEGEALKSPSGLKYDAIIHCATDIPWSKFSSNLSPNGKVIDLTPNFKSFLRSIFKKLTFSKQKLVPFLLSPNAEDLKLLGDLVKDGKLRTIVDSQYPLEKAEAAWVRSTEGHATGKVVVNHQDVSVG>Azfi_s0002.g001541MADQLMKAVQYSSYGGGHTALTHVQLPIPIPKKGEVLVKVEFASVNPIDWKIQSGALKPIMPPKFPYIPGTDVAGEVVAVGPGVDAFTTGDKVLSWIDLRVGGSLAEFAVVPLSSTVKRPLEVSAVDACCIPVAGLTALQAIKDYVGLKLDGKSDENLLITAASGGVGLYAVQIAKLTGAHVTATCGARNFELVRSLGADEILDYKTPEGAALQSPSGRKYDAVIHCARGIPWSTFSPVLKPAAKVVDLTPDLKSLATTALKKVTFCKQELLPFLMSGKAEDLEVLVALARDGKLKTIVDSRYPLARSGEAWARSIEGHSTGKVVVEVQG>XP_002975582.2 CEQORHMAEGASKKMMAALQYSKYGEGATGLKHVEIPVPTPKKDEILVKIEAAAINPVDWKLQKGMLKVIYSIKFPYIPCTDVSGEVVSVGPGVTGFSQGDKVISWLDLKRGGGFAQYAVAGIKYTAKRPSGVSAVEAAALPVAGLTAYQSLRDSGGLKYDGSYKGNVLITAASGGVGTYAVQLAKLAGAHVTATCGARNVDLVRSLGADEVLDYKTPEGAALKSPSGKKYDVVLQCATTMPWSTFQPVLSSKGKVIDLTPSFGVMLTSAFKFLTMSKQKMTIFVMSANAKDLGELAELVNEKKVKSLIDSEFPLDKAEDAWAKSIESHATGKIVVTNKDEAASY>MA_959201g0010 CEQORHVDVAGEVVSVGPGVNDLTTGDKIVSMLSQRSGGGLAEYVVAPIKTTVKRPSGVSAENGAGLPTAALSALQAIRDFAGVKLDGSGKEMNLLITAASGGVGLYAVQIAKLGRAHVTATXADEVLDYKTPEGAALKSPSGRKYDAVIHCTSNISWSTFQPNLSPNGKVIDLTPNFMSIATTFVKKLTLSRQQIVPLIVSGNSKDLALIVRLVNEGKVKTVIDSKYPLEKAADAWAKSMDGHATGKIVVGKEHGWSCHRKDCSNI>XP_002970156.2 CEQORHMEMQGSMPVIQYAKFGGDLESGHAPVPSPGPGELLVKVQAASLNPVDWKIQQGKYKTLNIPAQFPYIPCSDISGEVVSLGPGVTGFSVGDKVVSCLDHKRGGGLAKYASAEVRFTTRIPSQISPIEAAGLPIAGLTALYSLQEAAGIAIPSKDFQGTILVTAASGGVGTYAVQLAKLTGAHVVATCGSRNIDLIKSLGADEVLDYKTPQGAKLQTSSCSKFDVIIHCAYHRPPWSTFEHVLTRKGIVVDITPASESTTSSLKRKFDKAVKLITRQEAIGSLVPFYLTPHTSGIEALVDMMQKGKLKTIIDSVHPISNVTAAWFKCTEGHATGKIVVTL>XP_002978323.2 CEQORHMEMQGSMPVIQYVKFGGDLEKGHAPVPSPGPGELLVKVQAASLNPVDWKIQQGKYKTLNIPAQFPYIPCSDISGEVVSLGPGVTGFSVGDKVVSYLDHKRGGGLAKYASAEVRFTTRIPSQISPIEAAGLPIAGLTALYSLQEAAGIAIPSKDFQGTILVTAASGGVGTYAVQLAKLTGAHVVATCGSRNIDLIKSLGADEVLDYKTPQGAKLQSSSCSKFDVIIHCAYHRPPWSTFEHVLTRKGIVVDITPASESTTSSLKRKFDKAVKLITSQEAIGSLVPLYLTPHTSGIEALVDMMQKGKLKTIIDSVHPISNVTAAWFKCMEGHATGKIVVTL>Mp1g05710.1MRSAGGLRSASQWMRKTVGNMKAVVITQPGGPDVLQLKDVEEPSLGSSEVLIKVAATAVNRADTLQRQGKYPPPAGASLYPGLECSGVVEAVGDRVQRWKVGDEVCALLAGGGYAEKINVPEGQVLPVPKGVSLLHAAALPEVSCTVWSTVFMTSKLTAGESFLVHGGSSGIGTFAIQIAKAKGAKVFCTVGNQEKADCCRKLGADVVINYKEQDFVQVVKEETGNKGVNVILDHIGASYFQKNVDALSVDGRLFIIGFMGGAAGQVNLAPVMLKRLTVQGAGLRSRSLEQKSDIVAEVLKHVWPEVESGKVKPVVHSSFPLGDACKGHELLESSAHVGKIILTA>Azfi_s0211.g058133MKAVVVTALGGPEVLQVQEVPEPELKPGHVVVRIAASGVNRADTYQRHGTHPVPEGCPPYPGLECSGTIESVADDVQHWKVGDQVCALLGGGGYAEKVSLPAVHLLPIPKGVSLLDAAGFPEVACTVWSTVFMTSHLSGGESFLVHGGSSGIGTFAIQMAKYMGVRVFCTVGNEEKLQYCRSLGADVVINYKEDDFVKRVKEETGGKGVNVILDNIGASYLERNLDALSVDGRLFIIGFQGGAKAELNLGPILVKRLTIQAAGLRSRSVENKGQIVQEIRTHVWPAIESGKVKPVIYKSFPLAEAPDAHRLLESSRHIGKILLTT>XP_002960362.1 quinone oxidoreductase PIG3MRAVVAKGLGGPEVLELREVPDPDIGDDEVLIKVAAAGVNRADLLQLKGQHPPPPGAPPYLGLECSGVIERLGARVDGWKVGDEVCALLGGGGYAEKVAVAASQLLPIPRGVSLRDAASLPEVACTVWSTVFMACHLSAGESVLIHGGSSGIGTFAIQMAKSIGAQVLATAGSEKKLDLCKQLGAEVAINYKDDDFVARVKEATHGEGVDVILDMVGASYFERNLEALGMDGRLFIIGFQGGASGQLSLLPILKKRLIVSAAGLRTRAPESKAQIVAEVWQNVWPAVEEGKVKPVIYRVFPLEEAAQALQLMVANQHFGKILLTP>XP_002967353.1 quinone oxidoreductase PIG3MRAVVAKGLGGPEVLELREVPDPDIGDDEVLIKVAAAGVNRADLLQLKGQHPPPPGAPPYLGLECSGVIERLGARVDGWKVGDEVCALLGGGGYAEKVAVAASQLLPIPRGVSLRDAASLPEVACTVWSTVFMACHLSAGESVLIHGGSSGIGTFAIQMAKSIGAQVFATAGSEKKLDLCKQLGAEVAINYKEDDFVARVKEATHGEGVDVVLDMVGASYFERNLEALGMDGRLFIIGFQGGASGQLSLLPILKKRLIVSAAGLRTRAPESKAQIVAEVWQNVWPAVEERKVKPVIYRVFPLEEAAQALQLMVANQHFGKILLTP>AT4G21580.1MKAIVISEPGKPEVLQLRDVADPEVKDDEVLIRVLATALNRADTLQRLGLYNPPPGSSPYLGLECSGTIESVGKGVSRWKVGDQVCALLSGGGYAEKVSVPAGQIFPIPAGISLKDAAAFPEVACTVWSTVFMMGRLSVGESFLIHGGSSGIGTFAIQIAKHLGVRVFVTAGSDEKLAACKELGADVCINYKTEDFVAKVKAETDGKGVDVILDCIGAPYLQKNLDSLNFDGRLCIIGLMGGANAEIKLSSLLPKRLTVLGAALRPRSPENKAVVVREVEKNVWPAIEAGKVKPVIYKYLPLSQAAEGHSLMESSNHIGKILLET>Azfi_s0002.g004335MVLPHDMRFVDLPSPGAPEAMIVARGPLPVPKPGEILIRAEAIGVNRPDVAQRQGHYPPPADASPVLGLEVAGQVVALGDGAAGFAVGDKVCALANGGAYAEYCAVPASQALAWPQGYDAIRAAALPETFFTVWANLFMMAGLKEGESVLIHGGSSGIGTTAIQLAQALGATAFVTVGNAEKAEACFRLGAARAINYKSEDFAEVVKRETSGKGVDVILDMIGASYFDRNLACLARDGRLSIIAFLGGAVAEKANLSPIMVKRLRVMGSTMRPRSTQEKREIRDQLQAKVWPLLEAGTVAPVINRGRIKGRQAATCACLARCTDAVCGVRSRLPSRPRRSQRIAALRDDDDDIALDGQGAIARVGKIVGRQVLDEVVDVPALRQWKARGHLVLQRSCGVKITSAEFQQNVGLYQDAAQQAPVAITKNGRTHTVLMSAAMFELVTKGRLARRIEDLDQETLHAIASSAVPDEFSHLDDLIKDWQP>F39B2.3MSKSICKSSMRAAVVRRFGAPDVIEAVESDMPRLEKNQVLVRNYAAGVNPVDTYIRAGQYGKLPNLPYVPGKDGAGFVELVGESVKNVKVGDRVWYGSEADSTAEYVAVNRPFELPEGVSFEEGASLGVPYLTAYRALFHLAGAKTGDVILVHGASGGVGSALMQLAAWRNIEAVGTAGSADGIRFVKSLGARNVYNHSDKQYVSKMKNDYPGGFNHIFEMAAHTNLNTDLGLLAPRGRVAVIGNRAETTINARQLMVTEGAVYGVALGMSSEAELLDFGINIVSFLKETEFRPLINKLYRLEQLGLAHEEIMNNKGAKGNLVVQIEH>NP_001344601.1 quinone oxidoreductaseMATGQKLMRAIRVFEFGGPEVLKLQSDVVVPVPQSHQVLIKVHACGVNPVETYIRSGAYSRKPALPYTPGSDVAGIIESVGDKVSAFKKGDRVFCYSTVSGGYAEFALAADDTIYPLPETLNFRQGAALGIPYFTACRALFHSARARAGESVLVHGASGGVGLATCQIARAHGLKVLGTAGSEEGKKLVLQNGAHEVFNHKEANYIDKIKMSVGDKDKGVDVIIEMLANENLSNDLKLLSHGGRVVVVGCRGPIEINPRDTMAKETSIIGVSLSSSTKEEFQQFAGLLQAGIEKGWVKPVIGSEYPLEKAAQAHEDIIHGSGKTGKMILLL>NP_001123514.1 quinone oxidoreductase aMATGQKLMRAVRVFEFGGPEVLKLRSDIAVPIPKDHQVLIKVHACGVNPVETYIRSGTYSRKPLLPYTPGSDVAGVIEAVGDNASAFKKGDRVFTSSTISGGYAEYALAADHTVYKLPEKLDFKQGAAIGIPYFTAYRALIHSACVKAGESVLVHGASGGVGLAACQIARAYGLKILGTAGTEEGQKIVLQNGAHEVFNHREVNYIDKIKKYVGEKGIDIIIEMLANVNLSKDLSLLSHGGRVIVVGSRGTIEINPRDTMAKESSIIGVTLFSSTKEEFQQYAAALQAGMEIGWLKPVIGSQYPLEKVAEAHENIIHGSGATGKMILLL>NP_001123515.1 quinone oxidoreductase bMATGQKLMRAVRVFEFGGPEVLKLRSDIAVPIPKDHQVLIKVHACGVNPVETYIRSGTYSRKPLLPYTPGSDVAGVIEAVGDNASAFKKGDRVFTSSTISGGYAEYALAADHTVYKLPEKLDFKQGAAIGIPYFTAYRALIHSACVKAGESVLVHGASGGVGLAACQIARAYGLKILGTAGTEEGQKIVLQNGAHEVFNHREVNYIDKIKVVGSRGTIEINPRDTMAKESSIIGVTLFSSTKEEFQQYAAALQAGMEIGWLKPVIGSQYPLEKVAEAHENIIHGSGATGKMILLL>Mp3g08280.1 NADPH:quinone reductaseMERETIRALVVRSLGDPKTPLSDKNCPVSEMQWPRPALTSPTSVRVKVKVTSVNFSTVLQIQGLYQEKPKLPYVPGGDFSGVVTEAGAKVTHVKVGDRVCGFVNYGSFAEEFVADESEMFLIPSGCDLVAAGALPVAFGTSHIALDHRANLRPGQVLLVLGAGGGVGLAAVELGKLMGAIVIAVARGKEKVDLLRSTGADFVLDSSEDGIIKPVQAFLKSKKLRGVDVLYDPVGGKQHKEALKLVKWGGQILVIGFASGEIPSIPANIALVKNWTVHGLYWGSYVIHQPHILRDSMKQLLRWAAEGKLDVHISHQFPLSQANLAFATVMDRKAIGKVIFTLEDKPSRL>AT3G56460.1MEALVCRKLGDPTATNPGSPESPVEVSKTHPIPSLNSDTSVRVRVIATSLNYANYLQILGKYQEKPPLPFIPGSDYSGIVDAIGPAVTKFRVGDRVCSFADLGSFAQFIVADQSRLFLVPERCDMVAAAALPVAFGTSHVALVHRARLTSGQVLLVLGAAGGVGLAAVQIGKVCGAIVIAVARGTEKIQLLKSMGVDHVVDLGTENVISSVKEFIKTRKLKGVDVLYDPVGGKLTKESMKVLKWGAQILVIGFASGEIPVIPANIALVKNWTVHGLYWGSYRIHQPNVLEDSIKELLSWLSRGLITIHISHTYSLSQANLAFGDLKDRKAIGKVMIALDHKTALSSKL>NP_009602.1 NADPH:quinone reductaseMKCTIPEQQKVILIDEIGGYDVIKYEDYPVPSISEEELLIKNKYTGVNYIESYFRKGIYPCEKPYVLGREASGTVVAKGKGVTNFEVGDQVAYISNSTFAQYSKISSQGPVMKLPKGTSDEELKLYAAGLLQVLTALSFTNEAYHVKKGDYVLLFAAAGGVGLILNQLLKMKGAHTIAVASTDEKLKIAKEYGAEYLINASKEDILRQVLKFTNGKGVDASFDSVGKDTFEISLAALKRKGVFVSFGNASGLIPPFSITRLSPKNITLVRPQLYGYIADPEEWKYYSDEFFGLVNSKKLNIKIYKTYPLRDYRTAAADIESRKTVGKLVLEIPQ>Mp7g12910.1MVLGNGTATLVTGAGNGIGRGLSLSLASKGATVTVVEYSEADGLETVRLIEVEHAKLLKKPRAPSAIFIKCDVTVPEQLFRAFELHEQTYGRLDVCVNNAGIGEDQNFDADLSSDGKGKWRRTMDVNLSAVIDGTRLAVQTMRRTKQPGVIINVASAAGLYPSIAMPIYSASKAGVVMFSRSLGGLKREGIRVNALCPEFVDTALAKLVNPRVITNLGGYLPMDAILKGALQLIEDESKAGDTLWLTVRRGAEYWPTPEEKAKYELPRSRRIVRPLSKPIPPAIPDEYKKVIIHKLSSDFRTASKIVTVPLELPLKPGHVLVKNLYAGVNASDVNFSSGRYFGGKAKLPFDAGFEAVGVVASLGEGVDSNILVPGSPVATLTYGGFSEFCQVPFKNVIPMPVAVPELVALLTSGLTASLALEQAGRIKSGETVLVTAAAGGTGQFAVQLAKLAGNTVIATCGGEEKAKFLRSLGVDRVIDYKKESIKAVLKKEFPNGIDLIYESVGGEMFTTCLNALARRGRLIVIGMISQYQGENGWQPGNYPGLAEKILAKSQSVVGFFLNDYTRMWRDHAARLTKLYLDKKLKVTVDSKLFAGVEAIADAVEHLHSGQSLGKVVVRLAPETTNHAQARL>AT1G49670.2MEIKPGLSALVTGGASGIGRALCLALAEKGVFVTVADFSEEKGQETTSLVREANAKFHQGLSFPSAIFVKCDVTNRGDLLAAFDKHLATFGTLDICINNAGISTPLRFDKDDTDGSKSWKHTINVDLIAVVEGTQLAIKAMKAKQKPGVIINMGSAAGLYPMPVDPIYAASKAGVVLFTRSLAYYRRQGIRINVLCPEFIKTDLAEAIDASILESIGGYMSMDMLIKGAFELITDEKKAGACLWITKRRGLEYWPTPMEETKYLVGSSSRKRPSFKVSTKIEFPQSFEKMIVHTLSHKFRSATRIVRAPLQLPIGPHQVLLKIIYAGVNASDVNFSSGRYFTGGSPKLPFDAGFEGVGLIAAVGESVKNLEVGTPAAVMTFGAYSEYMIVSSKHVLPVPRPDPEVVAMLTSGLTALIALEKLYDILKLLVQLSLTFSLSYGNSQAGQMKSGETVLVTAAAGGTGQFAVQLAKLSGNKVIATCGGSEKAKLLKELGVDRVIDYKSENIKTVLKKEFPKGVNIIYESVGGQMFDMCLNALAVYGRLIVIGMISQYQGEKGWEPAKYPGLCEKILAKSQTVAGFFLVQYSQLWKQNLDKLFNLYALGKLKVGIDQKKFIGLNAVADAVEYLHSGKSTGKVVVCIDPAFEQKTSRL>NP_666202.2 prostaglandin reductase 3MLRLAAAGARAIVDMSYARHFLDFQGSAIPRTMQKLVVTRLSPNFHEAVTLRRDCPVPLPGDGDLLVRNRFVGINASDINYSAGRYDPSLKPPFDIGFEGIGEVVALGLSASARYTVGQAVAYMAPGSFAEYTVVPASIAIPMPSVKPEYLTMLVSGTTAYLSLEELGELSEGKKVLVTAAAGGTGQFAVQLSKIAKCHVIGTCSSDEKAAFLKSIGCDRPINYRTEPVETVLKQEYPEGVDVVYESVGGAMFDLAVDALATKGRLIVIGFISGYQSPTGLSPIKAGVLPTKLLKKSASLRGFFLNHYFSKYQAAMERLLELYARGDLVCEVDLGHLAPDGRFIGLESVFQAVDYMYTGKNTGKLVVELPHPVSSKL>NP_787103.1 prostaglandin reductase 3.1MLRLVPTGARAIVDMSYARHFLDFQGSAIPQAMQKLVVTRLSPNFREAVTLSRDCPVPLPGDGDLLVRNRFVGVNASDINYSAGRYDPSVKPPFDIGFEGIGEVVALGLSASARYTVGQAVAYMAPGSFAEYTVVPASIATPVPSVKPEYLTLLVSGTTAYISLKELGGLSEGKKVLVTAAAGGTGQFAMQLSKKAKCHVIGTCSSDEKSAFLKSLGCDRPINYKTEPVGTVLKQEYPEGVDVVYESVGGAMFDLAVDALATKGRLIVIGFISGYQTPTGLSPVKAGTLPAKLLKKSASVQGFFLNHYLSKYQAAMSHLLEMCVSGDLVCEVDLGDLSPEGRFTGLESIFRAVNYMYMGKNTGKIVVELPHSVNSKL>NP_001293022.1prostaglandin reductase 3.2MAPGSFAEYTVVPASIATPVPSVKPEYLTLLVSGTTAYISLKELGGLSEGKKVLVTAAAGGTGQFAMQLSKKAKCHVIGTCSSDEKSAFLKSLGCDRPINYKTEPVGTVLKQEYPEGVDVVYESVGGAMFDLAVDALATKGRLIVIGFISGYQTPTGLSPVKAGTLPAKLLKKSASVQGFFLNHYLSKYQAAMSHLLEMCVSGDLVCEVDLGDLSPEGRFTGLESIFRAVNYMYMGKNTGKIVVELPHSVNSKL>R04B5.5MSQDNLSAVLYGVDDLRLEQVPIPKPGPNQVLVKVHTVGICGSDVHYWTHGAIGPFVVKEPMIVGHETSGIVSEVGNEVKHLKVGDRIAMEPGLPCKLCEHCKTGRYNLCPEMRFFATPPVHGTLSRFVVHDADFCFKLPDNLSFEDGALIEPLSVAIHACRRGNVQMGHRVLVLGAGPIGVLNLITAKAVGAGKVVITDLDDGRLALAKKLGADATINVKGKSLDAVKSEIITALGDQQPDVCIECTGAQPSIETAITTTKSGGVIVLVGLGADRVEIPIIESATREVDMRGIFRYVNCYPTAIELISSGKLNLSGLTRAHYKLEETQEAFKRTQKADVIKVFIQC>R04B5.6MSQDNLSAVLYGINDLRLEQAPISKPGPRQVLVKINTVGICGSDVHFLTHGAIGSFVVKEPMVLGHESSGVVSEIGSEVKGFKVGDRIAMEPGLPCKLCEHCKIGRYNLCPDMRFFATPPVNGALSRFVVHDADFCFKLPDNLSFEDGALLEPLSVAIQACRRGTVQMGQKILVLGAGPIGVLNLLTAKAIGASKVVITDLNDERLALARLLGADATINVMGKRSDEVRSEIIKAFGDQQPHVSIECTGVQPCVETAIMTTRSGGVVVLVGLGAERVEIPLIQSPTREVDLRGTFRSANCYSTAIELISSGKLDLSGLTRAHYKLEESLEAFKRTQNGDVIKVFIHC>D2063.1MVSSDVPKTQRALIFESYGGPLEIKQLPIPQPNEDELLVKMEYSGICHSDVHTWLGDFHYVSKCPMIGGHEGAGSVISVGSKVKNWQIGDKVGIKLVQGNCLNCEYCQTGHEPLCPHVWNIGVQKYGTFQEYATIRDVDAIKIPKSMNMAAAAPVLCGGVTAYKALKESEVKSGQIVAVTGAGGGLGSFAIQYARAMGMRVVAIDHPSKEAHCKSLGAEWFVDAFGTEDIVAHIREITDGGAHGVVNFAAAKVPMEKALEYVRKRGTVVFVGLAKDSKILVDTIPLIFNAVKIKGSIVGSRLDVNEAMDFVARGAVNVPLELVKLEDVAEVYTKMHDGKINSRVVVDFSL>NP_009703.3 ADH5MPSQVIPEKQKAIVFYETDGKLEYKDVTVPEPKPNEILVHVKYSGVCHSDLHAWHGDWPFQLKFPLIGGHEGAGVVVKLGSNVKGWKVGDFAGIKWLNGTCMSCEYCEVGNESQCPYLDGTGFTHDGTFQEYATADAVQAAHIPPNVNLAEVAPILCAGITVYKALKRANVIPGQWVTISGACGGLGSLAIQYALAMGYRVIGIDGGNAKRKLFEQLGGEIFIDFTEEKDIVGAIIKATNGGSHGVINVSVSEAAIEASTRYCRPNGTVVLVGMPAHAYCNSDVFNQVVKSISIVGSCVGNRADTREALDFFARGLIKSPIHLAGLSDVPEIFAKMEKGEIVGRYVVETSK>NP_014555.1 ADH1MSIPETQKGVIFYESHGKLEYKDIPVPKPKANELLINVKYSGVCHTDLHAWHGDWPLPVKLPLVGGHEGAGVVVGMGENVKGWKIGDYAGIKWLNGSCMACEYCELGNESNCPHADLSGYTHDGSFQQYATADAVQAAHIPQGTDLAQVAPILCAGITVYKALKSANLMAGHWVAISGAAGGLGSLAVQYAKAMGYRVLGIDGGEGKEELFRSIGGEVFIDFTKEKDIVGAVLKATDGGAHGVINVSVSEAAIEASTRYVRANGTTVLVGMPAGAKCCSDVFNQVVKSISIVGSYVGNRADTREALDFFARGLVKSPIKVVGLSTLPEIYEKMEKGQIVGRYVVDTSK>Mp1g09240.1MSETAPNVTVSPKVDDTRLMKAVEWHGKKDVRVNSKRPMPLVTDPRDVVLQVTSSAICGSDLHLYLGNMLGMVPGDILGHEFMGVVHEVGPDVKSVKKGDRVVVCFDIGCGECYFCSKLQAFSCCGNTNPSQEEKAMYGDRTAGFFGYSHLTGGYPGGQAEYVRVPFADVNTLKVPENLSDEKVVLLSDILPTAWHANELGEVGQGDVVAIWGAGPVGILAAHCAQARGAKEVFLIDAVQYRLDFAKQKLPGLRTINFKQEKVYEALRKVASHGIDVGIDAVGMHYADSVLHKLQMMTMLETDTPEIVNDIVYNVRKGGRVSIVGAYAGYTNHFNLGAFMEKSLTMRGGQTPVQKYWKHLLELVQKSQLNPSLVITHQQPLEKAPELYKTFNDKVDNCIKVVMHPGLA>AT3G45770.1MAALMESVVGRALKFSSTANFRSIRRGETPTLCIKSFSTIMSPPSKAIVYEEHGSPDSVTRLVNLPPVEVKENDVCVKMIAAPINPSDINRIEGVYPVRPPVPAVGGYEGVGEVYAVGSNVNGFSPGDWVIPSPPSSGTWQTYVVKEESVWHKIDKECPMEYAATITVNPLTALRMLEDFVNLNSGDSVVQNGATSIVGQCVIQLARLRGISTINLIRDRAGSDEAREQLKALGADEVFSESQLNVKNVKSLLGNLPEPALGFNCVGGNAASLVLKYLREGGTMVTYGGMSKKPITVSTTSFIFKDLALRGFWLQSWLSMGKVKECREMIDYLLGLARDGKLKYETELVPFEEFPVALDKALGKLGRQPKQVITF>NP_013872.1 Yim1pMSDEIVTNKSVTYVNNTTPVTITSSELDLRSCYQDDEVVIEVHAAALNPIDFITHQLCNSYIFGKYPKTYSRDYSGVIIKAGKDVDNRWKVGDKVNGMYSHIYGERGTLTHYLILNPAKDVPITHMVEVPKDENDPYDDFVYAAAWPLTFGTAFSTLYDFKKDWTSDSKVLVIGASTSVSYAFVHIAKNYFNIGTVVGICSKNSIERNKKLGYDYLVPYDEGSIVENVKKLKQSVLENDKFDMIFDSVGNHDFFPVIDQFLKPKAKNSFYVTIAGNNKADYKNISWRDFVSLSSILKAINPFKKYNWRFGHPYPPNNFIEVGNEMIKKGTYKPPIDSVYEFDQYKEAIDRLMSNRAKGKVVVKMK>NP_009484.2 Ast1pMAKDILKNQDPKLQAMIVEHSAPAPKEIPMDAPVLKRVARPLRHVKFIPIKSLIFHTKTGPMDFSYEKKIKTPIPKNKIVVRVSNVGLNPVDMKIRNGYTSSIYGEIGLGREYSGVITEVGENLNYAWHVGDEVYGIYYHPHLAVGCLQSSILVDPKVDPILLRPESVSAEEAAGSLFCLATGYNILNKLSKNKYLKQDSNVLINGGTSSVGMFVIQLLKRHYKLQKKLVIVTSANGPQVLQEKFPDLADEMIFIDYLTCRGKSSKPLRKMLEEKKISQYDPVEDKETILNYNEGKFDVVLDFVGGYDILSHSSSLIHGGGAYVTTVGDYVANYKEDIFDSWDNPSANARKMFGSIIWSYNYTHYYFDPNAKTASANNDWIEQCGDFLKNGTVKCVVDKVYDWKDHKEAFSYMATQRAQGKLIMNVEKF>NP_598440.1 quinone oxidoreductase-like 1MKGLYFQQSSTNEEVTFVFQEKENVPVTEDNFVRVQVKACALSHINTKLLAEMKMEKDFFPVGREVSGIVLEVGRKVTFFQPDDEVVGILPLDSEDPGLCEVIRVHEHYLVHKPEKVSWTEAAGVIRDGVRACTALYYLSQLSPGKSVLIMDGASAFGTIAIQLAHHRGAKVISTAHSLEDKQHLERLRPSIARVIDVSNGKVHVAESCLEETGGLGVDIVIDAGVRLYSKDDEPAVKLHLPHKHDIITLLGVGGHWVTTEENLQLDPPDSHCLFLKGATVAFLNDEVWNLSNAQQGKYLCILKDVMEKLSAGVFRPLLDEPIPLYEAKVSMEVVQKNQERKKQVVQF>NP_665857.2 quinone oxidoreductase-like 1MKGLYFQQSSTDEEITFVFQEKEDLPVTEDNFVKLQVKACALSQINTKLLAEMKMKKDLFPVGREIAGIVLDVGSKVSFFQPDDEVVGILPLDSEDPGLCEVVRVHEHYLVHKPEKVTWTEAAGSIRDGVRAYTALHYLSHLSPGKSVLIMDGASAFGTIAIQLAHHRGAKVISTACSLEDKQCLERFRPPIARVIDVSNGKVHVAESCLEETGGLGVDIVLDAGVRLYSKDDEPAVKLQLLPHKHDIITLLGVGGHWVTTEENLQLDPPDSHCLFLKGATLAFLNDEVWNLSNVQQGKYLCILKDVMEKLSTGVFRPQLDEPIPLYEAKVSMEAVQKNQGRKKQVVQF>BRADI_5g06207v3MICSRAFFSSSIQESTQATLAQEVKIMGDPARPETMRAVQYSGYGGGAAALKYVEIPVPSLKKDEVLIKVEAASINPADCNIQKGLLRPFVPRFPFIPVSDVAGEIVEVGAAVSEFKVGDRVVSKLIFWKAGGLAEYVAASESVTVALPAGVSTVDAAGLPVAGLTALQAVRAIGTKFDGTGSGSNILITAGSGGIGTYAVQLAKLGNHNVTATCGARNLELVADMGADEVLDYKTPEGAALKNNSGKKYDYIVNTTNAGKWSAFKPALSSHGRVVDLAPNLANFVASMLTLFSKKKKLSTVLLSLGMEDLRFLLELVKEGKLKTVVDSRHPFDKAADAWEKSMSGHATGKVIVEM>BRADI_5g07080v3MASAATPTTTPAKMRAVQYDACGGGAAGLKHVEVPIPSAKKNEVLLRLEAATINPVDWKIQKGDLRPLLPRRLPFIPVTDVAGVVVDVGPGVKDFIAGDQVVAMLNSLNGGGLAEYAVASTNLTVKRTPEVSAADGAGLPIAAGTALQALRSIGAKFDGTGKPLNVLITAASGGVGLYAVQLAKLANLHVTATCGARNMDLVKSLGGDEVMDYKTPQGVSLQSPSGRKYDGVVHCTVGISWSVFKPLLSDTGRAIDITPNFSAILTSALHKVTFSKKRLVPLLLWPNKADLEFLVGLLKDGKLKTVIDSRFPLSDASKAWQSSIDGHATGKIVVEMESR>BRADI_4g34400v3MQKHHGARALPKIVVAGSTLFRRAAAKFTAMPSSTSSSSSPSRTMRAVLYDKYGGGAEGLKHVEVPIPSPKKGELLLRMEAASINRVDWRFQQGKARPILPSKFPFTPVCELAGEVVELGSGVSGFAAGDKVIAVNFPGGGGLAEYAVVSASCAATRPPEVSATEGACLPIAAATALAALRTAGVGLDARGPPKNVLVTAASGGVGTFAVQLAKLSGKHHVTVTSGARNLGLVRALGANDALDYGTPEGAALRRAGPAGRKHDAVVHCAEGFPWSAFEPALADAGGVVVDLTARVASVAVAALQRVLFARKRLVPLLVSPKKEDMEVLLGLVRRGQIRVVIDSRYPLSRAHEGWAKSMGGHATGKIVVEMGVTEGARTRRTSSSHLFVPLNLL>BRADI_2g50080v3 isoform 1MWWRAVARARRRVAGARPVSTAAGAEKSSRAVLVPRFGGPEVLELRQGVPVPDLKPGEVLVRARAVSINPLDLRMRSGYGRCIFEPLLPLILGRDISGEVAATGTSVSSFFIGQEVFGALHPTAMRGTCADYAILSQDELTPKPSMLTHVEASAIPFAALTAWRALHGTAGISEGQRVLVIGGGGAVGLSAVQLAVAAGCSVSATCGAQSIEQVLAAGAEKAIDYTSEDTESAVTGKFDAVLDTIGVPETERIGINLLRRGGHYMTLQGEAAALADRYGLYVGLPAATATLLKKQMQYRCSHGIEYWWTYMRADPEGLHEIRRLSGAGKLQIPVEKTFPISQVREAHEAKEKKLVPGKVVLEFD>BRADI_2g50080v3 isoform 2MWWRAVARARRRVAGARPVSTAAGAEKSSRAVLVPRFGGPEVLELRQGVPVPDLKPGEVLVRARAVSINPLDLREASAIPFAALTAWRALHGTAGISEGQRVLVIGGGGAVGLSAVQLAVAAGCSVSATCGAQSIEQVLAAGAEKAIDYTSEDTESAVTGKFDAVLDTIGVPETERIGINLLRRGGHYMTLQGEAAALADRYGLYVGLPAATATLLKKQMQYRCSHGIEYWWTYMRADPEGLHEIRRLSGAGKLQIPVEKTFPISQVREAHEAKEKKLVPGKVVLEFD##Fig3C_aligned##>Mp3g19030.1 MpRTN4IP1L-------------------------------------------------------------------------------------------------------------------------------------------------MRRQS-------------------------------------------------------------------------------------------------VQMFRSVEA-----------------------LPAQACIGSFRGLST--------CTAVLVRRFGG--PEVME---------YRNGVALPDL-GPNDVLVRARAVSVNPLDIRMRG-GYGKSL-F------------QPLLPIVL---------GRDVSGEISAVGSGVRQ--LQVGQEVFGALHP---------------------------------------------TAIRGTYSNYAILA------EEQLTAKPASLSHVD-----AAAIPFAALTAWRALR-----------------------STARIQNGQKVLVIGGGGAVGLAATYLAK-AAGC-H-VSV----TCGKKSVERVME----AGAQQA-VDYTSEN---IREQLK--D-------------------------RFDAVLDTIGMPETESLGINVLRK---------------------GGH---YLTLQG-EIVSMADKYGLLAGGAAAAAK-LVQKQVQYKQS------HGI-EYWWTVMRT-------DAEALEEIARLAKEGSLKVPVG------KT--FPLAEAAEAHKAREGKKTV--------------------------------------GKV--------VLEV--E-------------------------------------------------------------------------------------------------------->XP_024530385.1 RTN4IP1 homolog-------------------------------------------------------------------------------------------------------------------------------------------------MRRW----------------------------------------------------------------------------------------------------------------------------------LASPYCRGHSRSIST--------CRAVVLPRFGG--PEVLE---------LRKDVPVPHL-EAEEVLVRTVAAGVNPLDIKMRE-GYGRSL-F------------QPLLPIVL---------GRDVSGEIVAIGNGVKR--LHVGQHVFGALHP---------------------------------------------TAVRGTYSDYAILQ------EEQLALKPKSLTHVE-----AAAIPFAALTAWRALW-----------------------STARMKSGQKVLIMGGGGAVGLAAIQLAR-AAGC-S-VAA----TCGKRSCSRVGE----AGAELI-VDYTSEN---TREQLT--W-------------------------RFDAVLDTIGVETTEALGVNVLKR---------------------GGH---YMTLQG-EAVKLADKYGLIVGGGIATAT-LLRKRMQYTQS------HGI-DYSWIIMRT-------DAEGLEQIARLAKEGRLKIPVG------ET--FSLDEAAKAQTARENKDSQ--------------------------------------GKV--------VLQV--QQE------------------------------------------------------------------------------------------------------>AT3G15090.1-------------------------------------------------------------------------------------------------------------------------------------------------MRVM------------------------------------------------------------------------------------------------------RSLRGNSG-----------------AGLVFRPARLNSLRSVFTG-------CRAVILPRFGG--PEVFE---------LRENVPVPNL-NPNEVLVKAKAVSVNPLDCRIRA-GYGRSV-F------------QPHLPIIV---------GRDVSGEVAAIGTSVKS--LKVGQEVFGALHP---------------------------------------------TALRGTYTDYGILS------EDELTEKPSSISHVE-----ASAIPFAALTAWRALK-----------------------SNARITEGQRLLVFGGGGAVGFSAIQLAV-ASGC-H-VTA----SCVGQTKDRILA----AGAEQA-VDYTTED---IELAVK--G-------------------------KFDAVLDTIGGPETERIGINFLRK---------------------GGN---YMTLQG-EAASLTDKYGFVVGLPLATSL-LMKKKIQYQYS------HGI-DYWWTYMRA-------DPEGLAEIQRLVGAGKLKIPVE------KT--FPITDVVAAHEAKEKKQIP--------------------------------------GKV--------VLEF----------------------------------------------------------------------------------------------------------->BRADI_2g50080v3 isoform 1M--------------------------------------------------------------------------------------------------------------------------W--------------------WRAVA------------------------------------------------------------------------------------------------------RARRRVAG-----------------A----RP--VSTAAGAEKS-------SRAVLVPRFGG--PEVLE---------LRQGVPVPDL-KPGEVLVRARAVSINPLDLRMRS-GYGRCI-F------------EPLLPLIL---------GRDISGEVAATGTSVSS--FFIGQEVFGALHP---------------------------------------------TAMRGTCADYAILS------QDELTPKPSMLTHVE-----ASAIPFAALTAWRALH-----------------------GTAGISEGQRVLVIGGGGAVGLSAVQLAV-AAGC-S-VSA----TCGAQSIEQVLA----AGAEKA-IDYTSED---TESAVT--G-------------------------KFDAVLDTIGVPETERIGINLLRR---------------------GGH---YMTLQG-EAAALADRYGLYVGLPAATAT-LLKKQMQYRCS------HGI-EYWWTYMRA-------DPEGLHEIRRLSGAGKLQIPVE------KT--FPISQVREAHEAKEKKLVP--------------------------------------GKV--------VLEF--D-------------------------------------------------------------------------------------------------------->BRADI_2g50080v3 isoform 2M--------------------------------------------------------------------------------------------------------------------------W--------------------WRAVA------------------------------------------------------------------------------------------------------RARRRVAG-----------------A----RP--VSTAAGAEKS-------SRAVLVPRFGG--PEVLE---------LRQGVPVPDL-KPGEVLVRARAVSINPLDLR--------------------------------------------------------------------------------------------------------------------------------------------------------E-----ASAIPFAALTAWRALH-----------------------GTAGISEGQRVLVIGGGGAVGLSAVQLAV-AAGC-S-VSA----TCGAQSIEQVLA----AGAEKA-IDYTSED---TESAVT--G-------------------------KFDAVLDTIGVPETERIGINLLRR---------------------GGH---YMTLQG-EAAALADRYGLYVGLPAATAT-LLKKQMQYRCS------HGI-EYWWTYMRA-------DPEGLHEIRRLSGAGKLQIPVE------KT--FPISQVREAHEAKEKKLVP--------------------------------------GKV--------VLEF--D-------------------------------------------------------------------------------------------------------->F56H1.6 RAD-8M------------------------------------------------------------------------------------------------------------------------------------------------IEKMILR------------------------------------------------------------------------------------------------------------------------------------RFFSTKS-ST--------MRAWVSENGSG-----VE---------LKE-VPLPVINKPGQVLLKVKAASVNPIDVDMSQ-GYGREF-LGTWKKIESCDAAASRFPLIP---------GRDCTAVVESVGGDVHN--LAPGDEVMAVVPV----------------------------------------------ILPGTHAEFVVTD------SKYCSKKPSNLSFVS-----AAALPYVASTAYSAFT--------------------IARVSQRNAKQQRVLIHGGAGGVGSMAIQLLK-AWGC-EKIVA----TCAKGSFDIVKQ----LGAIP--VDYTSDQ---ATQELI--E-----------------------HAPFEVILDTVDS-QLAKWSDNVMGVWR-------------------NCV---HVSIVS-PLMREMDKNGVPLG--------LVTTAMKHFERSFQSHLRGR-WFSYAFFRP-------SSDLMSQLSRFAEDGKIVPVVE------QV--MGFEELEKAYEKVSQLNGR--------------------------------------GKT--------VIKY--D-------------------------------------------------------------------------------------------------------->NP_570962.2 RTN4IP1MGV---------------------------------------------------------------------------------------------LKTCV--------------------------------------------LRRSACAAA----------------------------------------------C--------------------------------------------------------------------FWRRTVIPKPPFRGISTTSARS-TV--------MPAWVIDKYGK--NEVLR---------FTQNMMLPIIHYPNEVIIKVHAASVNPIDVNMRS-GYGATA-LNMKRDPLHMKTKGEEFPLTL---------GRDVSGVVMECGLDVKY--FQPGDEVWAAVP----------------------------------------------PWKQGTLSEFVVVS------GNEVSHKPKSLTHTQ-----AASLPYVALTAWSAINK-------------------VGGLSDRNCKGKRALILGASGGVGTFAIQVMK-AWGA-H-VTA----VCSKDASELVRK----LGADEV-IDYTLGS---VEEQLK--SL-----------------------KLFDFILDNVGG-STETWALNFLKKWS-------------------GAT---YVTLVT-PFLLNMDRLGVADG--------MLQTGVTVGTKALKHLWQGV-HYRWAFFMA-------SGPYLDEIAELVDAGKIRPVIE------RT--FPFSEVPEAFLKVERGHAR--------------------------------------GKT--------VVNV--V-------------------------------------------------------------------------------------------------------->NP_116119.2 RTN4IP1.1MEF---------------------------------------------------------------------------------------------LKTCV--------------------------------------------LRRNACTAV----------------------------------------------C--------------------------------------------------------------------FWRSKVVQKPSVRRISTTSPRS-TV--------MPAWVIDKYGK--NEVLR---------FTQNMMMPIIHYPNEVIVKVHAASVNPIDVNMRS-GYGATA-LNMKRDPLHVKIKGEEFPLTL---------GRDVSGVVMECGLDVKY--FKPGDEVWAAVP----------------------------------------------PWKQGTLSEFVVVS------GNEVSHKPKSLTHTQ-----AASLPYVALTAWSAINK-------------------VGGLNDKNCTGKRVLILGASGGVGTFAIQVMK-AWDA-H-VTA----VCSQDASELVRK----LGADDV-IDYKSGS---VEEQLK--SL-----------------------KPFDFILDNVGG-STETWAPDFLKKWS-------------------GAT---YVTLVT-PFLLNMDRLGIADG--------MLQTGVTVGSKALKHFWKGV-HYRWAFFMA-------SGPCLDDIAELVDAGKIRPVIE------QT--FPFSKVPEAFLKVERGHAR--------------------------------------GKT--------VINV--V-------------------------------------------------------------------------------------------------------->NP_001305675.1 RTN4IP1.2-------------------------------------------------------------------------------------------------------------------------------------------------------------------------------------------------------------------------------------------------------------------------------------------------------------------------------------------------------------------------------------MKRDPLHVKIKGEEFPLTL---------GRDVSGVVMECGLDVKY--FKPGDEVWAAVP----------------------------------------------PWKQGTLSEFVVVS------GNEVSHKPKSLTHTQ-----AASLPYVALTAWSAINK-------------------VGGLNDKNCTGKRVLILGASGGVGTFAIQVMK-AWDA-H-VTA----VCSQDASELVRK----LGADDV-IDYKSGS---VEEQLK--SL-----------------------KPFDFILDNVGG-STETWAPDFLKKWS-------------------GAT---YVTLVT-PFLLNMDRLGIADG--------MLQTGVTVGSKALKHFWKGV-HYRWAFFMA-------SGPCLDDIAELVDAGKIRPVIE------QT--FPFSKVPEAFLKVERGHAR--------------------------------------GKT--------VINV--V-------------------------------------------------------------------------------------------------------->Mp6g05150.1-------------------------------------------------------------------------------------------------------------------------------------------------MAGVVTAGVGGNVAVLAGQ------------------------------------CDGRQRRRRSSSSCKA---------SMSLVPATKFG---------GAVGLRTKENGGRAWRRELNSSAVLGVRAEIRTEEASADSVAMAIP-QT--------QQAWVYQAYGE--KEVMS---------IGE-VAVPEL-NPDQVLIKVCAAALNPVDSKRRG-GKFQAT--------------DSDLPIVP---------GYDCSGIVVKVGSEVSK--FKVGDEVYGDTSEF------------------------------------CLN----GPKQYGTLAQYTAAE------EKLLAKKPQSLTFAE-----AASLPLALQTAYQGWQ------------------------KAGIKEGHKVMVLGGAGGVGTLAIQLAKQVFKA-SSVAA----TASTGKVDLLKS----LGADVV-VDYTKQK---LE-EWP--E-------------------------KYDVVYDTVGK----GEGTKVVKP---------------------DGG---LVVITG-PVEPPGFRYVIVS----------------------------------------------KGSDLEELNPYFETGSMQAVLD------PQGLFPFTKVVEAFGYLETGRAF--------------------------------------GKV--------VISP--IE------------------------------------------------------------------------------------------------------->AT1G23740.1 Alkenal/one oxidoreductase-------------------------------------------------------------------------------------------------------------------------------------------------MNAALATT-TATTPVLRRETP------------------------------LLHYC----SLTTKS-----------------------------------PVYQINRVRFGSCVQTVSKKFLKISASSQSASAA-VNVTADASIP-KE--------MKAWVYSDYGG--VDVLK---------LESNIVVPEI-KEDQVLIKVVAAALNPVDAKRRQ-GKFKAT--------------DSPLPTVP---------GYDVAGVVVKVGSAVKD--LKEGDEVYANVSEK------------------------------------ALE----GPKQFGSLAEYTAVE------EKLLALKPKNIDFAQ-----AAGLPLAIETADEGLV------------------------RTEFSAGKSILVLNGAGGVGSLVIQLAKHVYGA-SKVAA----TASTEKLELVRS----LGADLA-IDYTKEN---IE-DLP--D-------------------------KYDVVFDAIGM---CDKAVKVIKE---------------------GGK---VVALTG-AVTPPGFRFVVTS----------------------------------------------NGDVLKKLNPYIESGKVKPVVD------PKGPFPFSRVADAFSYLETNHAT--------------------------------------GKV--------VVYP--IP------------------------------------------------------------------------------------------------------->MA_10433694g0010 2-methylene-furan-3-one reductase-------------------------------------------------------------------------------------------------------------------------------------------------MDTFAATTITART------------------------------------------CINSFPRAGGAD---------------DIQSHVKFGKIGFGWNQRG--QSSYKLRHKSSNRTRGSRGVSVYAMADSAAAV-DQKTEKFVVP-SV--------QKAWIYKEYGAA-KDVLQ---------LDDGVPVPEV-KEDQVLIKVCAAALNPVDFKRRQ-GKFKAT--------------DSPLPTVP---------GYDVAGIVIKVGSKVEG--FKEGDEVYGDINEK------------------------------------ALD----NPKQYGSLGEYTAVE------EKLVALKPKNLDFTQ-----AASLPLAILTAYEGLE------------------------RAGFGPGKSILVLGGAGGVGSLVIQLARKVFGA-STIAA----TSSTGKLEFLKS----LGTDIS-IDYTKQN---FE-ELP--E-------------------------KYDVIYDAVGQ---CDKAVKAIKG---------------------GGS---VVALTG-AVTPPGFRFVVTS----------------------------------------------NGSVLSKLNPYLESGEIKPIID------PKGSFSFSQLVEAFSYLETCRAT--------------------------------------GKV--------VISP--IP------------------------------------------------------------------------------------------------------->XP_002971209.2 2-methylene-furan-3-one reductaseMHP-------------------------------------------------------------------------------------------------------------------------------DLA------------MESV---------------------------------------------------CVSQ-----------------------------------SPWFPVG--QLSRSKKRARATLTFASSSSKIG----------EMASTVAAIP-SL--------HKAWVYKEYGAA-KDVLK---------LEE-LPVPPL-APDQVLVKVHAAALNPVDFKRRL-GKFKAT--------------DSDLPTVP---------GYDVAGVVAKVGSEVSS--LKQGDEVYGDISEF------------------------------------ALD----KPKQWGSIAQYTAVE------EKLLAIKPRNLSFIQ-----AASLPLAIQTAQEGFD------------------------KAKLRAGQSVLVLGGAGGVGSLAIQLAKFVYGA-SLVAA----TTSTPKLELVKS----LGADIV-IDYKEKN---FE-DMP--E-------------------------RYDVVFDAVGE----PKGTNVVKE---------------------GGT---AVVLTG-PVNPPGFRFVVTS----------------------------------------------NGSNLVRLKDFLESGTVKPVID------PKGPFDFGSVVDAFCYLEGGRAS--------------------------------------GKV--------VISV--LQ------------------------------------------------------------------------------------------------------->XP_024543199.1 2-methylene-furan-3-one reductaseMHP-------------------------------------------------------------------------------------------------------------------------------DLA------------MESV---------------------------------------------------CVSQ-----------------------------------SPWFPVG--QLSRSKKRARATLTFASSSSKIG----------EMASTVAAIP-SL--------HKAWVYKEYGAA-KDVLK---------LEE-LPVPPL-APDQVLVKVHAAALNPVDFKRRL-GKFKAT--------------DSDLPTVP---------GYDVAGVVAKVGSEVSS--LKQGDEVYGDISEF------------------------------------ALD----KPKQWGSIAQYTAVE------EKLLAIKPRNLSFIQ-----AASLPLAIQTAQEGFD------------------------KAKLRAGQSVLVLGGAGGVGSLAIQLAKFVYGA-SLVAA----TTSTPKLGLVKS----LGADIV-IDYKEKN---FE-DMP--E-------------------------RYDVVFDAVGE----PKGTNVVKE---------------------GGT---AVVLTG-PVNPPGFRFVVTS----------------------------------------------NGSNLVRLKDFLESGTVKPVID------PKGPFDFGSVVDAFCYLEGGRAC--------------------------------------GKV--------VISV--LQ------------------------------------------------------------------------------------------------------->Azfi_s0003.g007918-------------------------------------------------------------------------------------------------------------------------------------------------MEFVVAISHCPRSPLVQ--------------------------------------CCSGIELRTSSIA------------NLNNRHNSFRASVNSHSHLINPTTSKTQLKKSSHYPPHGR------IHAKMISET-EVSSSVAEIP-AV--------QKAWFYEEYGD---DVLK---------FGE-VPVPQI-LPDQVLIKVHAAALNAVDSKRRT-GKFKAT--------------DSPLPTVP---------GYDVSGVIVKVGSEVTS--FKEGDEVYANISEA------------------------------------ALN----GPKQWGSIAQYTVTE------EKLLGIKPKNLSFAE-----AASLPLAIETALEGLE------------------------RAGFKEGQTLLVLGGAGGVGSLAIQLAKQVFGA-SLVAA----TTSTPKVEFVKS----LGADVV-IDYKKEK---FE-DLP--E-------------------------KYDVVFDGVGE---CEKAVKVVKE---------------------GGS---VIALTG-PVTPPGFRFVVTS----------------------------------------------KGSSLAKLNPYLESGKVKPILD------PKGQFSFSQVVEAFSYIGTGRAT--------------------------------------GKV--------VIAP--IE------------------------------------------------------------------------------------------------------->Azfi_s0123.g048404-------------------------------------------------------------------------------------------------------------------------------------------------------------------------------------------------------------------------------------------------------------------------------------------------MG-SL--------QKAWFYRDYGS--VEVLE---------YGD-VEVPQITSPDQVLIKVHAASLNPLDYKRRF-GYIKSP--------------TIPFPHVP---------GFDVAGVVVKVGSDVKK--FKEGDEVYGDIAEF------------------------------------AIA----APKQIGTLAQYTVTE------EKVLALKPKSLSFEE-----AASLPLALLTAQTGFE------------------------KAAFKPGQSVLILGGAGGVGTLAIQLAKHVYGA-SLVAT----TTSTGKLEFVKS----LGADVA-IDYTTTK---YY-ELP--E-------------------------KYDFVYDTVGD---GANAAKAVKE---------------------GGA---VLTIADHSAKPPVIAYGLIA----------------------------------------------NGDNLAKLNPFLEEGKLKPVID------PKGAFPFSQVKEAFEYLETSRAR--------------------------------------GKL--------VIAP--IH------------------------------------------------------------------------------------------------------->MA_427213g0010 2-methylene-furan-3-one reductase-------------------------------------------------------------------------------------------------------------------------------------------------------------------------------------------------------------------------------------------------------------------------------------------------MD-YM--------QKAWFYKEYGT--REVLQ---------YGD-FPVPQL-GDNQVLVRVRAAALNPVDFKLRN-GEIKDT--------------GLEFPVVP---------GCDVAGVIVDEGDNVSK--FSKGDEVYGNIQNF------------------------------------NTG----RPKQCGTLTQYTAVE------ESLIASKPENMSFEE-----AASFPLALQTAQEGFD------------------------RVNFQRGQSVFIVGGAGGVGSLAIQLAKYVYGA-SKIAT----TTSTGKVDFVRQ----LGADTV-IDYTKQS---YD-QIP--Q-------------------------KFDFVFDTVGD---SHRSYVVAKE---------------------KAK---IIDIANPSPHPRAEFFIVTA----------------------------------------------SGSNLERLRGYIECEKLKPVID------PISPYSFSNVMEAFKHLESGRAR--------------------------------------GKI--------VISP--ID------------------------------------------------------------------------------------------------------->MA_207070g0010 2-methylene-furan-3-one reductase-------------------------------------------------------------------------------------------------------------------------------------------------------------------------------------------------------------------------------------------------------------------------------------------------MD-YM--------QKAWFYNEYGP--RAVLQ---------YGD-FPVPQL-GDTQVLVRVRAAALNPVDFKLRN-GVFKDT--------------GLEFPVVP---------GCDVAGVIVDEGDNVSM--FSKGDEVYGNIQSF------------------------------------NTV----GPKQYGTLAQYTAVE------ESLIALKPENMSFEE-----AASFPLALQTAQEGFD------------------------RAKFLRGQTVFIVGGEGGVGSLAIQLAKYVYGA-SKIAT----TTSPGKVDFVKK----LGPDMV-IDYTKKS---YD-QIL--E-------------------------KFDFVFDTIGD---SHRSYVVAKE---------------------EGK---IIDIANSSPHPRAEFYIVTP----------------------------------------------SGSNLERLRSYIECEKLKPVID------PTGPYAFSNVMEAFKHLESGRAR--------------------------------------GKV--------VISP--ID------------------------------------------------------------------------------------------------------->MA_8441g0010 2-methylene-furan-3-one reductase-------------------------------------------------------------------------------------------------------------------------------------------------------------------------------------------------------------------------------------------------------------------------------------------------MS-KL--------QKAWFYNEYGS--IDVLQ---------FGE-VPVPTP-GPGQLLVKIRAAALNPVDFKRRD-GLFRNK--------------DSDFPAVP---------GCDAAGVVVEVGDGVSK--FKNGDEVYSDIQDF------------------------------------GAG----RPKQWGTLAQYTVVE------EYLVAPKPTNLSFEE-----AASLPLALLTAQQAFD------------------------IAKFEKGKSVFIVGGAGGVGSLAIQLARHVYGA-SKIVS----TASTGKLDFVKS----LGADLV-IDYTKQS---YD-QIS--E-------------------------KFDFVFDTIGD---SAKSHVVAKE---------------------EAK---ILDIASFQPTSRVEFFGVSP----------------------------------------------HAKNLEKLQPYIESKKLKPVID------PKSPYSFSDVIEAFKHQESGRAR--------------------------------------GKI--------VISP--IE------------------------------------------------------------------------------------------------------->MA_10436234g0010 2-methylene-furan-3-one reductase-------------------------------------------------------------------------------------------------------------------------------------------------------------------------------------------------------------------------------------------------------------------------------------------------MS-KL--------QKAWFYNEYGS--IDVLQ---------FGE-VPVPTP-GPGQLLVKIRAAALNPVDFKRRD-GRIRIA--------------DSDFPTVP---------GCDAAGVVVDVGDGVSK--FKNGDEVYSDIQNF------------------------------------VVG----KRKQWGTLAQYTVVE------EYLVAPKPTNLSFEE-----AASLPLALLTAQQAFD------------------------IAKFEKGKSVFIVGGAGGVGSLAIQLARHVYGA-SKIVS----TASTGKLDFVKS----LGADLV-IDYTKQS---YD-QIS--E-------------------------KFDFVFDTIGD---SAKSHVVAKE---------------------EAK---ILDIASFQPTSRVEFFGVSP----------------------------------------------HAKNLEKLQPYIESKKLKPVID------PKSPYSFSDVIEAFKHQESGRAR--------------------------------------GKI--------VISP--IE------------------------------------------------------------------------------------------------------->Mp1g20380.1 CEQORH-------------------------------------------------------------------------------------------------------------------------------------------------------------------------------------------------------------------------------------------------------------------------------------------------MA-EM--------MQAVQYSSYGGA-HAALQ---------HKE-IPIPVP-KKGEILVKVEAASVNPVDWKMQG-GVMRPM-L------------PAKFPHTP---------GTDIAGEVVKVGSEVKD--FAPGDKVVAWLEL----------------------------------------------KHGGAMAQYAVAN------PKTTVVRPPEVSAVE-----AACLPVAALTALQALTS-------------------GGMNFDGSYSG-NVLVTGASGGVGTYAVQLGK-IAGA-H-VTA----TCGERNIELIKF----LGADEV-LDYKTPE---GK-KLI--SPS---------------------GKKYDLVVNAAASAVTFSDMQPQLAP---------------------KGV---VFELTP-SPKTFLT---------------SVIKRVSMSKQ----------KYNQLMLNV-------EARNLEMLVGFVKEGKLHAVID------SK--FPLAKVEEAWKKSKEGHAV--------------------------------------GKI--------VITV--TEE------------------------------------------------------------------------------------------------------>AT4G13010 CEQORH------------------------------------------------------------------------------------------------------------------------------------------------------------------------------------------------------------------------------------------------------------------------------------------------MAG-KL--------MHALQYNSYGGG-AAGLE---------HVQ-VPVPTP-KSNEVCLKLEATSLNPVDWKIQK-GMIRPF-L------------PRKFPCIP---------ATDVAGEVVEVGSGVKN--FKAGDKVVAVLSH----------------------------------------------LGGGGLAEFAVAT------EKLTVKRPQEVGAAE-----AAALPVAGLTALQALTNP------------------AGLKLDGTGKKANILVTAASGGVGHYAVQLAK-LANA-H-VTA----TCGARNIEFVKS----LGADEV-LDYKTPE---GA-ALK--SPS---------------------GKKYDAVVHCANG-IPFSVFEPNLSE---------------------NGK---VIDITP-GPNAMWT---------------YAVKKITMSKK----------QLVPLLLIP-------KAENLEFMVNLVKEGKVKTVID------SK--HPLSKAEDAWAKSIDGHAT--------------------------------------GKI--------IVEP----------------------------------------------------------------------------------------------------------->BRADI_5g07080v3------------------------------------------------------------------------------------------------------------------------------------------------------------------------------------------------------------------------------------------------------------------------------------------------MAS-AATPTTTPAKMRAVQYDACGGG-AAGLK---------HVE-VPIPSA-KKNEVLLRLEAATINPVDWKIQK-GDLRPL-L------------PRRLPFIP---------VTDVAGVVVDVGPGVKD--FIAGDQVVAMLNS----------------------------------------------LNGGGLAEYAVAS------TNLTVKRTPEVSAAD-----GAGLPIAAGTALQALRS-------------------IGAKFDGTGKPLNVLITAASGGVGLYAVQLAK-LANL-H-VTA----TCGARNMDLVKS----LGGDEV-MDYKTPQ---GV-SLQ--SPS---------------------GRKYDGVVHCTVG-ISWSVFKPLLSD---------------------TGR---AIDITP-NFSAILT---------------SALHKVTFSKK----------RLVPLLLWP-------NKADLEFLVGLLKDGKLKTVID------SR--FPLSDASKAWQSSIDGHAT--------------------------------------GKI--------VVEM--ESR------------------------------------------------------------------------------------------------------>Azfi_s0035.g025558-----------------------------------------------------------------------------------------------------------------------------------------------------------------------------------------------------------------------------------------------------------------------------------------------MASS-KI--------MQALQYSSYGGG-ASSLK---------HVE-IPVPTP-KSGEILVKIEAASVNPVDWKIQS-GIARPF-I------------PFKFPYVP---------GTDIAGEVVSVGPGVNT--FSVGDKVISWLEL----------------------------------------------NKGGAFAEFAVAS------LKYTAKIPEGVTAID-----GCSLPVAAITALQAIRDAN----------------GAGIKLDGTSKA-NLLITAASGGVGIYAVQIAK-LTGA-H-VTA----TCGARNIDLVKN----LGANEV-LDYKTPE---GD-ALK--SPS---------------------GVKYDAIIHCATG-IPWSKFSSTLTP---------------------NGK---VIDLTP-NFKSIVH---------------TIFKKVTFSKQ----------KLVPFMASP-------NSEDLKLLGELVKDGKLRNIVD------SQ--HPFAKAEESWVRSMEGHAT--------------------------------------GKV--------VVTF--QE------------------------------------------------------------------------------------------------------->Azfi_s0035.g025557-------------------------------------------------------------------------------------------------------------------------------------------------------------------------------------------------------------------------------------------------------------------------------------------------------------------MNVY------------------------------------------VRKRDFAAQK-DLTFRQ-V------------VRKIIYVSVISFLAGYAGTDIAGEVVSVGPGVKT--FSVGDKIMSFLDL----------------------------------------------MKGGSLAEFAIAI------PKFTVKRPEGVSAID-----GCSLPVAGMSALQAIRNSN----------------GAGIKLDGASKA-NLLITAASGGVGTYALQIAK-LTGA-H-VTG----TCGARNIELVKS----LGADEI-LDYKTPE---GE-ALK--SPS---------------------GLKYDAIIHCATD-IPWSKFSSNLSP---------------------NGK---VIDLTP-NFKSFLR---------------SIFKKLTFSKQ----------KLVPFLLSP-------NAEDLKLLGDLVKDGKLRTIVD------SQ--YPLEKAEAAWVRSTEGHAT--------------------------------------GKV--------VVNH--QDVSVG--------------------------------------------------------------------------------------------------->Azfi_s0002.g001541------------------------------------------------------------------------------------------------------------------------------------------------------------------------------------------------------------------------------------------------------------------------------------------------MAD-QL--------MKAVQYSSYGGG-HTALT---------HVQ-LPIPIP-KKGEVLVKVEFASVNPIDWKIQS-GALKPI-M------------PPKFPYIP---------GTDVAGEVVAVGPGVDA--FTTGDKVLSWIDL----------------------------------------------RVGGSLAEFAVVP------LSSTVKRPLEVSAVD-----ACCIPVAGLTALQAIKDY------------------VGLKLDGKSDE-NLLITAASGGVGLYAVQIAK-LTGA-H-VTA----TCGARNFELVRS----LGADEI-LDYKTPE---GA-ALQ--SPS---------------------GRKYDAVIHCARG-IPWSTFSPVLKP---------------------AAK---VVDLTP-DLKSLAT---------------TALKKVTFCKQ----------ELLPFLMSG-------KAEDLEVLVALARDGKLKTIVD------SR--YPLARSGEAWARSIEGHST--------------------------------------GKV--------VVEV--QG------------------------------------------------------------------------------------------------------->XP_002975582.2 CEQORH-------------------------------------------------------------------------------------------------------------------------------------------------MAE-------------------------------------------------------------------------------------------------------------------------------------------GASK-KM--------MAALQYSKYGEG-ATGLK---------HVE-IPVPTP-KKDEILVKIEAAAINPVDWKLQK-GMLKVI-Y------------SIKFPYIP---------CTDVSGEVVSVGPGVTG--FSQGDKVISWLDL----------------------------------------------KRGGGFAQYAVAG------IKYTAKRPSGVSAVE-----AAALPVAGLTAYQSLRDS------------------GGLKYDGSYKG-NVLITAASGGVGTYAVQLAK-LAGA-H-VTA----TCGARNVDLVRS----LGADEV-LDYKTPE---GA-ALK--SPS---------------------GKKYDVVLQCATT-MPWSTFQPVLSS---------------------KGK---VIDLTP-SFGVMLT---------------SAFKFLTMSKQ----------KMTIFVMSA-------NAKDLGELAELVNEKKVKSLID------SE--FPLDKAEDAWAKSIESHAT--------------------------------------GKI--------VVTN--KDEAASY-------------------------------------------------------------------------------------------------->MA_959201g0010 CEQORH------------------------------------------------------------------------------------------------------------------------------------------------------------------------------------------------------------------------------------------------------------------------------------------------------------------------------------------------------------------------------------------------------------------VDVAGEVVSVGPGVND--LTTGDKIVSMLSQ----------------------------------------------RSGGGLAEYVVAP------IKTTVKRPSGVSAEN-----GAGLPTAALSALQAIRDF------------------AGVKLDGSGKEMNLLITAASGGVGLYAVQIAK-LGRA-H-VTA----T----------------XADEV-LDYKTPE---GA-ALK--SPS---------------------GRKYDAVIHCTSN-ISWSTFQPNLSP---------------------NGK---VIDLTP-NFMSIAT---------------TFVKKLTLSRQ----------QIVPLIVSG-------NSKDLALIVRLVNEGKVKTVID------SK--YPLEKAADAWAKSMDGHAT--------------------------------------GKI--------VVGK--EHGWSCH----------------------------------------------------------------RKD----------CSNI----------------->BRADI_4g34400v3MQKHHGARALPKIVVAGS---------------------------------TLFRRAAAKF-----------------------------------------------------------------------------TAMPSSTSSS-------------------------------------------------------------------------------------------------------------------------------------------SSPS-RT--------MRAVLYDKYGGG-AEGLK---------HVE-VPIPSP-KKGELLLRMEAASINRVDWRFQQ-GKARPI-L------------PSKFPFTP---------VCELAGEVVELGSGVSG--FAAGDKVIA-VNF----------------------------------------------PGGGGLAEYAVVS------ASCAATRPPEVSATE-----GACLPIAAATALAALRT-------------------AGVGLDARGPPKNVLVTAASGGVGTFAVQLAK-LSGKHH-VTV----TSGARNLGLVRA----LGANDA-LDYGTPE---GA-ALRRAGPA---------------------GRKHDAVVHCAEG-FPWSAFEPALAD--------------------AGGV---VVDLTA-RVASVAV---------------AALQRVLFARK----------RLVPLLVSP-------KKEDMEVLLGLVRRGQIRVVID------SR--YPLSRAHEGWAKSMGGHAT--------------------------------------GKI--------VVEMGVTEGARTR----------------------------------------------------------------RTS----------SSHL----FVPLNLL------>BRADI_5g06207v3---MICSRAF------FS---------------------------------SSIQESTQAT-----------------------------------------------------------------------------LAQEVKIMGD-------------------------------------------------------------------------------------------------------------------------------------------PARP-ET--------MRAVQYSGYGGG-AAALK---------YVE-IPVPSL-KKDEVLIKVEAASINPADCNIQK-GLLRPF-V------------P-RFPFIP---------VSDVAGEIVEVGAAVSE--FKVGDRVVSKLIF----------------------------------------------WKAGGLAEYVAAS------ESVTVALPAGVSTVD-----AAGLPVAGLTALQAVRA-------------------IGTKFDGTGSGSNILITAGSGGIGTYAVQLAK-LGNH-N-VTA----TCGARNLELVAD----MGADEV-LDYKTPE---GA-ALK--NNS---------------------GKKYDYIVNTTNA-GKWSAFKPALSS---------------------HGR---VVDLAP-NLANFVA---------------SMLTLFS-KKK----------KLSTVLLSL-------GMEDLRFLLELVKEGKLKTVVD------SR--HPFDKAADAWEKSMSGHAT--------------------------------------GKV--------IVEM----------------------------------------------------------------------------------------------------------->XP_002970156.2 CEQORH-----------------------------------------------------------------------------------------------------------------------------------------------------------------------------------------------------------------------------------------------------------------------------------------------MEMQ-GS--------MPVIQYAKFGG----DLE---------SGH-APVPSP-GPGELLVKVQAASLNPVDWKIQQ-GKYKTLNI------------PAQFPYIP---------CSDISGEVVSLGPGVTG--FSVGDKVVSCLDH----------------------------------------------KRGGGLAKYASAE------VRFTTRIPSQISPIE-----AAGLPIAGLTALYSLQEA------------------AGIAIPSKDFQGTILVTAASGGVGTYAVQLAK-LTGA-H-VVA----TCGSRNIDLIKS----LGADEV-LDYKTPQ---GA-KLQ--TSS---------------------CSKFDVIIHCAYHRPPWSTFEHVLTR---------------------KGI---VVDITP-ASESTTS---------------SLKRKFDKAVKLIT-RQEAIGSLVPFYLTP-------HTSGIEALVDMMQKGKLKTIID------SV--HPISNVTAAWFKCTEGHAT--------------------------------------GKI--------VVTL----------------------------------------------------------------------------------------------------------->XP_002978323.2 CEQORH-----------------------------------------------------------------------------------------------------------------------------------------------------------------------------------------------------------------------------------------------------------------------------------------------MEMQ-GS--------MPVIQYVKFGG----DLE---------KGH-APVPSP-GPGELLVKVQAASLNPVDWKIQQ-GKYKTLNI------------PAQFPYIP---------CSDISGEVVSLGPGVTG--FSVGDKVVSYLDH----------------------------------------------KRGGGLAKYASAE------VRFTTRIPSQISPIE-----AAGLPIAGLTALYSLQEA------------------AGIAIPSKDFQGTILVTAASGGVGTYAVQLAK-LTGA-H-VVA----TCGSRNIDLIKS----LGADEV-LDYKTPQ---GA-KLQ--SSS---------------------CSKFDVIIHCAYHRPPWSTFEHVLTR---------------------KGI---VVDITP-ASESTTS---------------SLKRKFDKAVKLIT-SQEAIGSLVPLYLTP-------HTSGIEALVDMMQKGKLKTIID------SV--HPISNVTAAWFKCMEGHAT--------------------------------------GKI--------VVTL----------------------------------------------------------------------------------------------------------->Mp1g05710.1MRSAGGL------------------------------------------------------------------------------------------------------------------------------------RSASQWMRKTV----------------------------------------------------------------------------------------------------------------------------------------------GN--------MKAVVITQPGG--PDVLQ---------LKD-VEEPSL-GSSEVLIKVAATAVNRADTLQRQ-GKYPP---------------PAGASLYP---------GLECSGVVEAVGDRVQR--WKVGDEVCALLAG-------------------------------------------------GGYAEKINVP------EGQVLPVPKGVSLLH-----AAALPEVSCTVWSTVF-----------------------MTSKLTAGESFLVHGGSSGIGTFAIQIAK-AKGA-KVFCT----VGNQEKADCCRK----LGADVV-INYKEQD---FVQVVK--EETG--------------------NKGVNVILDHIGA-SYFQKNVDALSV---------------------DGR---LFIIGF-MGGAAGQ-VNLAP---------VMLKRLTVQGA----------GLRSRSLEQK---SDIVAEVLKHVWPEVESGKVKPVVH------SS--FPLGDACKGHELLESSAHV--------------------------------------GKI--------ILTA----------------------------------------------------------------------------------------------------------->Azfi_s0211.g058133--------------------------------------------------------------------------------------------------------------------------------------------------------------------------------------------------------------------------------------------------------------------------------------------------------------MKAVVVTALGG--PEVLQ---------VQE-VPEPEL-KPGHVVVRIAASGVNRADTYQRH-GTHPV---------------PEGCPPYP---------GLECSGTIESVADDVQH--WKVGDQVCALLGG-------------------------------------------------GGYAEKVSLP------AVHLLPIPKGVSLLD-----AAGFPEVACTVWSTVF-----------------------MTSHLSGGESFLVHGGSSGIGTFAIQMAK-YMGV-RVFCT----VGNEEKLQYCRS----LGADVV-INYKEDD---FVKRVK--EETG--------------------GKGVNVILDNIGA-SYLERNLDALSV---------------------DGR---LFIIGF-QGGAKAE-LNLGP---------ILVKRLTIQAA----------GLRSRSVENK---GQIVQEIRTHVWPAIESGKVKPVIY------KS--FPLAEAPDAHRLLESSRHI--------------------------------------GKI--------LLTT----------------------------------------------------------------------------------------------------------->XP_002960362.1 quinone oxidoreductase PIG3--------------------------------------------------------------------------------------------------------------------------------------------------------------------------------------------------------------------------------------------------------------------------------------------------------------MRAVVAKGLGG--PEVLE---------LRE-VPDPDI-GDDEVLIKVAAAGVNRADLLQLK-GQHPP---------------PPGAPPYL---------GLECSGVIERLGARVDG--WKVGDEVCALLGG-------------------------------------------------GGYAEKVAVA------ASQLLPIPRGVSLRD-----AASLPEVACTVWSTVF-----------------------MACHLSAGESVLIHGGSSGIGTFAIQMAK-SIGA-QVLAT----AGSEKKLDLCKQ----LGAEVA-INYKDDD---FVARVK--EATH--------------------GEGVDVILDMVGA-SYFERNLEALGM---------------------DGR---LFIIGF-QGGASGQ-LSLLP---------ILKKRLIVSAA----------GLRTRAPESK---AQIVAEVWQNVWPAVEEGKVKPVIY------RV--FPLEEAAQALQLMVANQHF--------------------------------------GKI--------LLTP----------------------------------------------------------------------------------------------------------->XP_002967353.1 quinone oxidoreductase PIG3--------------------------------------------------------------------------------------------------------------------------------------------------------------------------------------------------------------------------------------------------------------------------------------------------------------MRAVVAKGLGG--PEVLE---------LRE-VPDPDI-GDDEVLIKVAAAGVNRADLLQLK-GQHPP---------------PPGAPPYL---------GLECSGVIERLGARVDG--WKVGDEVCALLGG-------------------------------------------------GGYAEKVAVA------ASQLLPIPRGVSLRD-----AASLPEVACTVWSTVF-----------------------MACHLSAGESVLIHGGSSGIGTFAIQMAK-SIGA-QVFAT----AGSEKKLDLCKQ----LGAEVA-INYKEDD---FVARVK--EATH--------------------GEGVDVVLDMVGA-SYFERNLEALGM---------------------DGR---LFIIGF-QGGASGQ-LSLLP---------ILKKRLIVSAA----------GLRTRAPESK---AQIVAEVWQNVWPAVEERKVKPVIY------RV--FPLEEAAQALQLMVANQHF--------------------------------------GKI--------LLTP----------------------------------------------------------------------------------------------------------->AT4G21580.1--------------------------------------------------------------------------------------------------------------------------------------------------------------------------------------------------------------------------------------------------------------------------------------------------------------MKAIVISEPGK--PEVLQ---------LRD-VADPEV-KDDEVLIRVLATALNRADTLQRL-GLYNP---------------PPGSSPYL---------GLECSGTIESVGKGVSR--WKVGDQVCALLSG-------------------------------------------------GGYAEKVSVP------AGQIFPIPAGISLKD-----AAAFPEVACTVWSTVF-----------------------MMGRLSVGESFLIHGGSSGIGTFAIQIAK-HLGV-RVFVT----AGSDEKLAACKE----LGADVC-INYKTED---FVAKVK--AETD--------------------GKGVDVILDCIGA-PYLQKNLDSLNF---------------------DGR---LCIIGL-MGGANAE-IKLSS---------LLPKRLTVLGA----------ALRPRSPENK---AVVVREVEKNVWPAIEAGKVKPVIY------KY--LPLSQAAEGHSLMESSNHI--------------------------------------GKI--------LLET----------------------------------------------------------------------------------------------------------->Azfi_s0002.g004335-------------------------------------------------------------------------------------------------------------------------------------------------M----------------------------------------------------------------------------------------------------------------------------------------------VLP-HD--------MRFVDLPSPGA--PEAMI---------VAR-GPLPVP-KPGEILIRAEAIGVNRPDVAQRQ-GHYPP---------------PADASPVL---------GLEVAGQVVALGDGAAG--FAVGDKVCALANG-------------------------------------------------GAYAEYCAVP------ASQALAWPQGYDAIR-----AAALPETFFTVWANLF-----------------------MMAGLKEGESVLIHGGSSGIGTTAIQLAQ-ALGA-TAFVT----VGNAEKAEACFR----LGAARA-INYKSED---FAEVVK--RETS--------------------GKGVDVILDMIGA-SYFDRNLACLAR---------------------DGR---LSIIAF-LGGAVAEKANLSP---------IMVKRLRVMGS----------TMRPRSTQEK---REIRDQLQAKVWPLLEAGTVAPVIN------RGR-IKGRQAATCACLARCTDAVCGVRSRLPSRPRRSQRIAALRDDDDDIALDGQGAIARVGKIVGRQVLDEVVDV--PALRQWKARGHLVLQRSCGVKITSAEFQQNVGLYQDAAQQAPVAITKNGRTHTVLMSAAMFELVTKGRLARRIEDLDQETLHAIASSAVPDEFSHLDDLIKDWQP>F39B2.3-------------------------------------------------------------------------------------------------------------------------------------------------MSKSICK--------------------------------------------------------------------------------------------------------------------------------------------SS--------MRAAVVRRFGA--PDVIE---------AVE-SDMPRL-EKNQVLVRNYAAGVNPVDTYIRA-GQYGK---------------LPNLPYVP---------GKDGAGFVELVGESVKN--VKVGDRVWYGSEA-------------------------------------------------DSTAEYVAVN--------RPFELPEGVSFEE-----GASLGVPYLTAYRALF-----------------------HLAGAKTGDVILVHGASGGVGSALMQLAA-WRNI-EAVGT----AGSADGIRFVKS----LGARNV-YNHSDKQ---YVSKMK--NDY---------------------PGGFNHIFEMAAH-TNLNTDLGLLAP---------------------RGR---VAVIGN-RAETT---INARQ---------LMVTEGAVYGV----------ALGMSSEAE-------LLDFGINIVSFLKETEFRPLIN------KL--YRLEQLGLAHEEIMNNKGAK-------------------------------------GNL--------VVQI--EH------------------------------------------------------------------------------------------------------->NP_001344601.1 quinone oxidoreductase-------------------------------------------------------------------------------------------------------------------------------------------------MATGQ----------------------------------------------------------------------------------------------------------------------------------------------KL--------MRAIRVFEFGG--PEVLK---------LQSDVVVPVP-QSHQVLIKVHACGVNPVETYIRS-GAYSR---------------KPALPYTP---------GSDVAGIIESVGDKVSA--FKKGDRVFCY------------------------------------------------STVSGGYAEFALAA------DDTIYPLPETLNFRQ-----GAALGIPYFTACRALF-----------------------HSARARAGESVLVHGASGGVGLATCQIAR-AHGL-KVLGT----AGSEEGKKLVLQ----NGAHEV-FNHKEAN---YIDKIK--MSVGDK------------------DKGVDVIIEMLAN-ENLSNDLKLLSH---------------------GGR---VVVVGC-RGPIE---INPRD---------TMAKETSIIGV----------SLSSSTKEE-------FQQFAGLLQAGIEKGWVKPVIG------SE--YPLEKAAQAHEDIIHGSGKT-------------------------------------GKM--------ILLL----------------------------------------------------------------------------------------------------------->NP_001123514.1 quinone oxidoreductase a-------------------------------------------------------------------------------------------------------------------------------------------------MATGQ----------------------------------------------------------------------------------------------------------------------------------------------KL--------MRAVRVFEFGG--PEVLK---------LRSDIAVPIP-KDHQVLIKVHACGVNPVETYIRS-GTYSR---------------KPLLPYTP---------GSDVAGVIEAVGDNASA--FKKGDRVFTS------------------------------------------------STISGGYAEYALAA------DHTVYKLPEKLDFKQ-----GAAIGIPYFTAYRALI-----------------------HSACVKAGESVLVHGASGGVGLAACQIAR-AYGL-KILGT----AGTEEGQKIVLQ----NGAHEV-FNHREVN---YIDKIK--KYVG--------------------EKGIDIIIEMLAN-VNLSKDLSLLSH---------------------GGR---VIVVGS-RGTIE---INPRD---------TMAKESSIIGV----------TLFSSTKEE-------FQQYAAALQAGMEIGWLKPVIG------SQ--YPLEKVAEAHENIIHGSGAT-------------------------------------GKM--------ILLL----------------------------------------------------------------------------------------------------------->NP_001123515.1 quinone oxidoreductase b-------------------------------------------------------------------------------------------------------------------------------------------------MATGQ----------------------------------------------------------------------------------------------------------------------------------------------KL--------MRAVRVFEFGG--PEVLK---------LRSDIAVPIP-KDHQVLIKVHACGVNPVETYIRS-GTYSR---------------KPLLPYTP---------GSDVAGVIEAVGDNASA--FKKGDRVFTS------------------------------------------------STISGGYAEYALAA------DHTVYKLPEKLDFKQ-----GAAIGIPYFTAYRALI-----------------------HSACVKAGESVLVHGASGGVGLAACQIAR-AYGL-KILGT----AGTEEGQKIVLQ----NGAHEV-FNHREVN---YIDKIK---------------------------------------------------------------------------------VVGS-RGTIE---INPRD---------TMAKESSIIGV----------TLFSSTKEE-------FQQYAAALQAGMEIGWLKPVIG------SQ--YPLEKVAEAHENIIHGSGAT-------------------------------------GKM--------ILLL----------------------------------------------------------------------------------------------------------->Mp3g08280.1 NADPH:quinone reductase-------------------------------------------------------------------------------------------------------------------------------------------------MER------------------------------------------------------------------------------------------------------------------------------------------------ET--------IRALVVRSLGD--PKTPL--SDKNCPVSEMQWPRPALTSPTSVRVKVKVTSVNFSTVLQIQ-GLYQE---------------KPKLPYVP---------GGDFSGVVTEAGAKVTH--VKVGDRVCGFVNY-------------------------------------------------GSFAEEFVAD------ESEMFLIPSGCDLVA-----AGALPVAFGTSHIALD-----------------------HRANLRPGQVLLVLGAGGGVGLAAVELGK-LMGA-IVIAV----ARGKEKVDLLRS----TGADFV-LDSSEDG---IIKPVQ--AFLKSKK-----------------LRGVDVLYDPVGG-KQHKEALKLVKW---------------------GGQ---ILVIGF-ASGEIPS-IPANI---------ALVKNWTVHGL----------YWGSYVIHQ----PHILRDSMKQLLRWAAEGKLDVHIS------HQ--FPLSQANLAFATVMDRKAI--------------------------------------GKV--------IFTL--EDKPS------------------------------------------------------------------RL-------------------------------->AT3G56460.1--------------------------------------------------------------------------------------------------------------------------------------------------------------------------------------------------------------------------------------------------------------------------------------------------------------MEALVCRKLGD--PTATNPGSPESPVEVSKTHPIPSLNSDTSVRVRVIATSLNYANYLQIL-GKYQE---------------KPPLPFIP---------GSDYSGIVDAIGPAVTK--FRVGDRVCSFADL-------------------------------------------------GSFAQFIVAD------QSRLFLVPERCDMVA-----AAALPVAFGTSHVALV-----------------------HRARLTSGQVLLVLGAAGGVGLAAVQIGK-VCGA-IVIAV----ARGTEKIQLLKS----MGVDHV-VDLGTEN---VISSVK--EFIKTRK-----------------LKGVDVLYDPVGG-KLTKESMKVLKW---------------------GAQ---ILVIGF-ASGEIPV-IPANI---------ALVKNWTVHGL----------YWGSYRIHQ----PNVLEDSIKELLSWLSRGLITIHIS------HT--YSLSQANLAFGDLKDRKAI--------------------------------------GKV--------MIAL--DHKTA---------------------------------------------------------------LSSKL-------------------------------->NP_009602.1 NADPH:quinone reductase-------------------------------------------------------------------------------------------------------------------------------------------------MK-----------------------------------------------------C---------------------------------------------------------------------------------------TIP-EQ--------QKVILIDEIGG--YDVIK---------YED-YPVPSI-SEEELLIKNKYTGVNYIESYFRK-GIY-----------------PCEKPYVL---------GREASGTVVAKGKGVTN--FEVGDQVA--------------------------------------------------YISNSTFAQYSKISS-----QGPVMKLPKGTSDEE--LKLYAAGLLQVLTALSFTN-----------------------EAYHVKKGDYVLLFAAAGGVGLILNQLLK-MKGA-HTIAV----ASTDEKLKIAKE----YGAEYL-INASKED---ILRQVL--KFTN--------------------GKGVDASFDSVGK-DTFEISLAALKR---------------------KGV---FVSFGN-ASGLIPP-FSITR---------LSPKNITLVRP----------QLYGYIADP-----EEWKYYSDEFFGLVNSKKLNIKIY------KT--YPLRDYRTAAADIESRKTV--------------------------------------GKL--------VLEI--PQ------------------------------------------------------------------------------------------------------->Mp7g12910.1MVLGNGTATL----VTGAGNGIGRGLSLSLASKGATVTVVEYSEADGLETVRLIEVEHAKLLKKPRAPSAIFIKCDVTVPEQLFRAFELHEQTYGRLDVCVNNAGIGEDQNFDADLSSDGKGKWRRTMDVNLSAVIDGTRLAVQTMRRTKQPGVIINVASAAGLYPSIAMPIYSASKAGVVMFSRSLGGLKREGIRVNALCPEFVDTALAKLVNPRVITNLGGYLPMDAILKGAL-QLIEDESKAGDTLWLTVRRGAEYWPTPEEK-AKYELPRSRRIVRPLSKPIPPAIP-DE--------YKKVIIHKLSSDFRTASK---------IVT-VPLELPLKPGHVLVKNLYAGVNASDVNFSS-GRYFGG--------------KAKLPFDA---------GFEAVGVVASLGEGVDSNILVPGSPVA--------------------------------------------------TLTYGGFSEFCQVP------FKNVIPMPV--AVPE-----LVALLTSGLTASLALE-----------------------QAGRIKSGETVLVTAAAGGTGQFAVQLAK-LAGN-TVIAT----CGGEEKAKFLRS----LGVDRV-IDYKKES---IKAVLK--KEF---------------------PNGIDLIYESVGG-EMFTTCLNALAR---------------------RGR---LIVIGM-ISQYQGE-NGWQPGNYPGLAEKILAKSQSVVGF----------FLNDYTRM--------WRDHAARLTKLYLDKKLKVTVD------SKLFAGVEAIADAVEHLHSGQSL--------------------------------------GKV--------VVRL--APETTNH-------------------------------------------------------------AQARL-------------------------------->AT1G49670.2MEIKPGLSAL----VTGGASGIGRALCLALAEKGVFVTVADFSEEKGQETTSLVREANAKFHQGLSFPSAIFVKCDVTNRGDLLAAFDKHLATFGTLDICINNAGISTPLRFDKD-DTDGSKSWKHTINVDLIAVVEGTQLAIKAMKAKQKPGVIINMGSAAGLYPMPVDPIYAASKAGVVLFTRSLAYYRRQGIRINVLCPEFIKTDLAEAIDASILESIGGYMSMDMLIKGAF-ELITDEKKAGACLWITKRRGLEYWPTPMEE-TKYLVGSSSRKRPSFKVSTKIEFP-QS--------FEKMIVHTLSHKFRSATR---------IVR-APLQLPIGPHQVLLKIIYAGVNASDVNFSS-GRYFTG-------------GSPKLPFDA---------GFEGVGLIAAVGESVKN--LEVGTPAA--------------------------------------------------VMTFGAYSEYMIVS------SKHVLPVPR--PDPE-----VVAMLTSGLTALIALEKLYDILKLLVQLSLTFSLSYGNSQAGQMKSGETVLVTAAAGGTGQFAVQLAK-LSGN-KVIAT----CGGSEKAKLLKE----LGVDRV-IDYKSEN---IKTVLK--KEF---------------------PKGVNIIYESVGG-QMFDMCLNALAV---------------------YGR---LIVIGM-ISQYQGE-KGWEPAKYPGLCEKILAKSQTVAGF----------FLVQYSQL--------WKQNLDKLFNLYALGKLKVGID------QKKFIGLNAVADAVEYLHSGKST--------------------------------------GKV--------VVCI--DPA-----------------------FE---------------------------------------QKTSRL-------------------------------->NP_666202.2 prostaglandin reductase 3-------------------------------------------------------------------------------------------------------------------------------------------------MLRLAAAG-----------------------ARAIVDMSYARHFLDFQG---------------------------------------------------------------------------------------------SAIP-RT--------MQKLVVTRLSPNFHEAVT---------LRRDCPVPLP-GDGDLLVRNRFVGINASDINYSA-GRYDP---------------SLKPPFDI---------GFEGIGEVVALGLSASAR-YTVGQAVA--------------------------------------------------YMAPGSFAEYTVVP------ASIAIPMPS--VKPE-----YLTMLVSGTTAYLSLE-----------------------ELGELSEGKKVLVTAAAGGTGQFAVQLSK-IAKC-HVIGT----CSSDEKAAFLKS----IGCDRP-INYRTEP---VETVLK--QEY---------------------PEGVDVVYESVGG-AMFDLAVDALAT---------------------KGR---LIVIGF-ISGYQSP-TGLSPIKAGVLPTKLLKKSASLRGF----------FLNHYFSK--------YQAAMERLLELYARGDLVCEVDLGHLAPDGRFIGLESVFQAVDYMYTGKNT--------------------------------------GKL--------VVEL--PHP-----------------------------------------------------------------VSSKL-------------------------------->NP_787103.1 prostaglandin reductase 3.1-------------------------------------------------------------------------------------------------------------------------------------------------MLRLVPTG-----------------------ARAIVDMSYARHFLDFQG---------------------------------------------------------------------------------------------SAIP-QA--------MQKLVVTRLSPNFREAVT---------LSRDCPVPLP-GDGDLLVRNRFVGVNASDINYSA-GRYDP---------------SVKPPFDI---------GFEGIGEVVALGLSASAR-YTVGQAVA--------------------------------------------------YMAPGSFAEYTVVP------ASIATPVPS--VKPE-----YLTLLVSGTTAYISLK-----------------------ELGGLSEGKKVLVTAAAGGTGQFAMQLSK-KAKC-HVIGT----CSSDEKSAFLKS----LGCDRP-INYKTEP---VGTVLK--QEY---------------------PEGVDVVYESVGG-AMFDLAVDALAT---------------------KGR---LIVIGF-ISGYQTP-TGLSPVKAGTLPAKLLKKSASVQGF----------FLNHYLSK--------YQAAMSHLLEMCVSGDLVCEVDLGDLSPEGRFTGLESIFRAVNYMYMGKNT--------------------------------------GKI--------VVEL--PHS-----------------------------------------------------------------VNSKL-------------------------------->NP_001293022.1prostaglandin reductase 3.2-----------------------------------------------------------------------------------------------------------------------------------------------------------------------------------------------------------------------------------------------------------------------------------------------------------------------------------------------------------------------------------------------------------------------------------------------------------------------------------------------MAPGSFAEYTVVP------ASIATPVPS--VKPE-----YLTLLVSGTTAYISLK-----------------------ELGGLSEGKKVLVTAAAGGTGQFAMQLSK-KAKC-HVIGT----CSSDEKSAFLKS----LGCDRP-INYKTEP---VGTVLK--QEY---------------------PEGVDVVYESVGG-AMFDLAVDALAT---------------------KGR---LIVIGF-ISGYQTP-TGLSPVKAGTLPAKLLKKSASVQGF----------FLNHYLSK--------YQAAMSHLLEMCVSGDLVCEVDLGDLSPEGRFTGLESIFRAVNYMYMGKNT--------------------------------------GKI--------VVEL--PHS-----------------------------------------------------------------VNSKL-------------------------------->R04B5.5-------------------------------------------------------------------------------------------------------------------------------------------------MSQDN--------------------------------------------------------------------------------------------------------------------------------------------------------LSAVLYG------VDDLR---------LEQ-VPIPKP-GPNQVLVKVHTVGICGSDVHYWTHGAIGPF--------------VVKEPMIV---------GHETSGIVSEVGNEVKH--LKVGDRIAMEPGLP-CKLCEHCKTGRYNLC-----PEMR-----------FFA----TPPVHGTLSRFVVHD------ADFCFKLPDNLSFED-----GALIE-PLSVAIHACR------------------------RGNVQMGHRVLVLGA-GPIGVLNLITAK-AVGAGKVVIT----DLDDGRLALAKK----LGADAT-INVKGKSLDAVKSEII--TALG--------------------DQQPDVCIECTGAQPSIETAITTTKS---------------------GGV---IVLVGL-GADRVE--IPIIE---------SATREVDMRGIFR------------------------YVNCYPTAIELISSGKLNLSGL------TRAHYKLEETQEAFKRTQKADVI---------------------------------------KV--------FIQC----------------------------------------------------------------------------------------------------------->R04B5.6-------------------------------------------------------------------------------------------------------------------------------------------------MSQDN--------------------------------------------------------------------------------------------------------------------------------------------------------LSAVLYG------INDLR---------LEQ-APISKP-GPRQVLVKINTVGICGSDVHFLTHGAIGSF--------------VVKEPMVL---------GHESSGVVSEIGSEVKG--FKVGDRIAMEPGLP-CKLCEHCKIGRYNLC-----PDMR-----------FFA----TPPVNGALSRFVVHD------ADFCFKLPDNLSFED-----GALLE-PLSVAIQACR------------------------RGTVQMGQKILVLGA-GPIGVLNLLTAK-AIGASKVVIT----DLNDERLALARL----LGADAT-INVMGKRSDEVRSEII--KAFG--------------------DQQPHVSIECTGVQPCVETAIMTTRS---------------------GGV---VVLVGL-GAERVE--IPLIQ---------SPTREVDLRGTFR------------------------SANCYSTAIELISSGKLDLSGL------TRAHYKLEESLEAFKRTQNGDVI---------------------------------------KV--------FIHC----------------------------------------------------------------------------------------------------------->CD2063.1-------------------------------------------------------------------------------------------------------------------------------------------------M-------------------------------------------------------------------------------------------------------------------------------------------VSSDVP-KT--------QRALIFESYGG----PLE---------IKQ-LPIPQP-NEDELLVKMEYSGICHSDVHTWL-GDFHY---------------VSKCPMIG---------GHEGAGSVISVGSKVKN--WQIGDKVGIKLVQGNCLNCEYCQTGHEPLC-----PHVW-----------NIG-----VQKYGTFQEYATIR------DVDAIKIPKSMNMAA-----AAPVLCGGVTAYKALK------------------------ESEVKSGQIVAVTGAGGGLGSFAIQYAR-AMGM-RVVAI-----DHPSKEAHCKS----LGAEWF-VDAFGTE--DIVAHIR--EITD--------------------GGAHGVVNFAAAK-VPMEKALEYVRK---------------------RGT---VVFVGL-AKDSKIL-VDTIP---------LIFNAVKIKGSIVG-----------------------SRLDVNEAMDFVARGAVNVPLEL---------VKLEDVAEVYTKMHDGKIN--------------------------------------SRV--------VVDF--SL------------------------------------------------------------------------------------------------------->NP_009703.3 ADH5-------------------------------------------------------------------------------------------------------------------------------------------------M-------------------------------------------------------------------------------------------------------------------------------------------PSQVIP-EK--------QKAIVFYETDG----KLE---------YKD-VTVPEP-KPNEILVHVKYSGVCHSDLHAWH-GDWPF---------------QLKFPLIG---------GHEGAGVVVKLGSNVKG--WKVGDFAGIKWLNGTCMSCEYCEVGNESQC-----PYLD-----------GTG-----FTHDGTFQEYATAD------AVQAAHIPPNVNLAE-----VAPILCAGITVYKALK------------------------RANVIPGQWVTISGACGGLGSLAIQYAL-AMGY-RVIGI----DGGNAKRKLFEQ----LGGEIF-IDFTEEK--DIVGAII--KATN--------------------GGSHGVINVSVSE-AAIEASTRYCRP---------------------NGT---VVLVGM-PAHAYCN-SDVFN---------QVVKSISIVGSCVG-----------------------NRADTREALDFFARGLIKSPIHL---------AGLSDVPEIFAKMEKGEIV--------------------------------------GRY--------VVET--SK------------------------------------------------------------------------------------------------------->NP_014555.1 ADH1-------------------------------------------------------------------------------------------------------------------------------------------------M----------------------------------------------------------------------------------------------------------------------------------------------SIP-ET--------QKGVIFYESHG----KLE---------YKD-IPVPKP-KANELLINVKYSGVCHTDLHAWH-GDWPL---------------PVKLPLVG---------GHEGAGVVVGMGENVKG--WKIGDYAGIKWLNGSCMACEYCELGNESNC-----PHAD-----------LSG-----YTHDGSFQQYATAD------AVQAAHIPQGTDLAQ-----VAPILCAGITVYKALK------------------------SANLMAGHWVAISGAAGGLGSLAVQYAK-AMGY-RVLGI----DGGEGKEELFRS----IGGEVF-IDFTKEK--DIVGAVL--KATD--------------------GGAHGVINVSVSE-AAIEASTRYVRA---------------------NGT---TVLVGM-PAGAKCC-SDVFN---------QVVKSISIVGSYVG-----------------------NRADTREALDFFARGLVKSPIKV---------VGLSTLPEIYEKMEKGQIV--------------------------------------GRY--------VVDT--SK------------------------------------------------------------------------------------------------------->Mp1g09240.1-------------------------------------------------------------------------------------------------------------------------------------------------MSETA-----------------------------------------------------------------------------------------------------------------------------------PNVTVSPKVDDTRL-------MKAVEWHG-----KKDVR---------VNSKRPMPLVTDPRDVVLQVTSSAICGSDLHLYL-GNMLGM-----------------VPGDIL--------GHEFMGVVHEVGPDVKS--VKKGDRVVVCFDIG-CGECYFCSKLQAFSCCGNTNPSQEEKAMYGDRTAGFFGYSHLTGGYPGGQAEYVRVP----FADVNTLKVPENLSDEK-----VVLLSDILPTAWHANE------------------------LGEVGQGDVVAIWGA-GPVGILAAHCAQ-ARGAKEVFLI----DAVQYRLDFAKQK---LPGLRT-INFKQEK---VYEALR--KVA---------------------SHGIDVGIDAVGM-HYADSVLHKLQMMTMLETDTPEIVNDIVYNVRKGGR---VSIVGA-YAGYTNH-FNLGA---------FMEKSLTMRGG-----------------------QTPVQKYWKHLLELVQKSQLNPSLVI----THQ--QPLEKAPELYKTFNDKVDNC-------------------------------------IKV--------VMHP--GLA------------------------------------------------------------------------------------------------------>AT3G45770.1---------------------------------------------------------------------------------------------------------------------------------------------MAALMESVVGRA-----------------------------------------------------LKFSS------------------------------------TANFRSIRRGE-------------------TPTLCIKSFSTIMS-PP--------SKAIVYEEHGS--PDSVT--------RLVN-LPPVEV-KENDVCVKMIAAPINPSDINRIE-GVYPV---------------RPPVPAVG---------GYEGVGEVYAVGSNVNG--FSPGDWVI---------------------------PS---------------------PPSSGTWQTYVVKE------ESVWHKIDKECPMEY-----AATITVNPLTALRMLE-----------------------DFVNLNSGDSVVQNGATSIVGQCVIQLAR-LRGI-STINLIRDRAGSDEAREQLKA----LGADEV-FSESQLN---VK-NVK--SLLGN-------------------LPEPALGFNCVGG-NAASLVLKYLRE---------------------GGT---MVTYGG-MSKKPIT-VSTTS---------FIFKDLALRGF----------WLQSWLSMGK---VKECREMIDYLLGLARDGKLKYETEL---------VPFEEFPVALD-----KAL--------------------------------------GKLG-RQP-KQVITF----------------------------------------------------------------------------------------------------------->NP_013872.1 Yim1p-------------------------------------------------------------------------------------------------------------------------------------------------MSDEI----------------------------------------------------------------------------------------------------------------------------------------------VT--------NKSVTYVNNTT--PVTIT---------SSE-LDLRSCYQDDEVVIEVHAAALNPIDFITHQ-LCNSYI--------------FGKYPKTY---------SRDYSGVIIKAGKDVDNR-WKVGDKVNGMYSHI--------------------------------------------YGERGTLTHYLILNPAKDVPITHMVEVPKDENDPYDDFVYAAAWPLTFGTAFSTLYD----------------------FKKDWTSDSKVLVIGASTSVSYAFVHIAKNYFNI-GTVVG----ICSKNSIERNKK----LGYDYL-VPYDEGS---IVENVK--KLKQSVL----------------ENDKFDMIFDSVGNHDFFPVIDQFLKP---------------------KAKNSFYVTIAG-NNKADYKNISWRD---------FVSLSSILKAI------NPFKKYNWRFGHP-----YPPNNFIEVGNEMIKKGTYKPPID------SV--YEFDQYKEAIDRLMSNRAK--------------------------------------GKV--------VVKM--K-------------------------------------------------------------------------------------------------------->NP_009484.2 Ast1p-------------------------------------------------------------------------------------------------------------------------------------------------MAKDILKNQDPKLQAMIVEHSAPAP-------------------------------------------------------------------------------------------------KEIPMDAPVLKRVARPLRHVKFIP-----------IKSLIFHTKTG--PMDFS---------YEKKIKTPIP--KNKIVVRVSNVGLNPVDMKIRN-GYTSSI-YG---------------EIGL---------GREYSGVITEVGENLNYA-WHVGDEVYGIYYHP--------------------------------------------HLAVGCLQSSILVDPK----VDPILLRPESVSAEE-----AAGSLFCLATGYNILNKLS--------------------KNKYLKQDSNVLINGGTSSVGMFVIQLLKRHYKLQKKLVI----VTSANGPQVLQEKFPDLADEMIFIDYLTCR-GKSSKPLR--KMLEEKKISQYDPVEDKETILNYNEGKFDVVLDFVGGYDILSHSSSLIHG---------------------GGA--YVTTVGDYVANYKEDIFDSWDN--------PSANARKMFGSIIWSY-----NYTHYYFDPNAKTASANNDWIEQCGDFLKNGTVKCVVD------KV--YDWKDHKEAFSYMATQRAQ--------------------------------------GKL--------IMNV--EKF------------------------------------------------------------------------------------------------------>NP_598440.1 quinone oxidoreductase-like 1--------------------------------------------------------------------------------------------------------------------------------------------------------------------------------------------------------------------------------------------------------------------------------------------------------------MKGLYFQQSST--NEEVT-------FVFQEKENVPVT-EDNFVRVQVKACALSHINTKLLA----------------EMKMEKDFFPV-----------GREVSGIVLEVGRKVTF--FQPDDEVVGILPL---------------------------------------------DSEDPGLCEVIRVH------EHYLVHKPEKVSWTE-----AAGVIRDGVRACTALY-----------------------YLSQLSPGKSVLIMDGASAFGTIAIQLAH-HRGA-KVIST----AHSLEDKQHLERLR--PSIARV-IDVSNGK-VHVAESCL--EETG--------------------GLGVDIVIDAGVR-LYSKDDEPAVKL-----H-LPH-----------KHDIITLLGVGG-HWVTTEENLQLDP---------PDSHCLFLKGATVAFLNDEVWNLSNAQQGK-------YLCILKDVMEKLSAGVFRPLLD------EP--IPLYEAKVSMEVVQKNQER--------------------------------------KKQ--------VVQF----------------------------------------------------------------------------------------------------------->NP_665857.2 quinone oxidoreductase-like 1--------------------------------------------------------------------------------------------------------------------------------------------------------------------------------------------------------------------------------------------------------------------------------------------------------------MKGLYFQQSST--DEEIT-------FVFQEKEDLPVT-EDNFVKLQVKACALSQINTKLLA----------------EMKMKKDLFPV-----------GREIAGIVLDVGSKVSF--FQPDDEVVGILPL---------------------------------------------DSEDPGLCEVVRVH------EHYLVHKPEKVTWTE-----AAGSIRDGVRAYTALH-----------------------YLSHLSPGKSVLIMDGASAFGTIAIQLAH-HRGA-KVIST----ACSLEDKQCLERFR--PPIARV-IDVSNGK-VHVAESCL--EETG--------------------GLGVDIVLDAGVR-LYSKDDEPAVKL-----QLLPH-----------KHDIITLLGVGG-HWVTTEENLQLDP---------PDSHCLFLKGATLAFLNDEVWNLSNVQQGK-------YLCILKDVMEKLSTGVFRPQLD------EP--IPLYEAKVSMEAVQKNQGR--------------------------------------KKQ--------VVQF-----------------------------------------------------------------------------------------------------------##Fig3C_aligned_trimmed##>Mp3g19030.1_MpRTN4IP1LRDVSGEISAVGSGVRQLQVGQEVFGTYSNYAILAQLTAKPASHVDAAAIPFAALTAWRALRTARIQNGKVLVIGGGAVGLAATYLAKAAGCHVSVTAQQVDYTSENIRQLKDRFDAVLDTIGMSLGINVLRKGGHYLTLQGEIVSMDAEALEEIARLAKEGSLKVPVGFPLAEAAEAHKKTVKVVLEV>XP_024530385.1_RTN4IP1 homologRDVSGEIVAIGNGVKRLHVGQHVFGTYSDYAILQQLALKPKTHVEAAAIPFAALTAWRALWTARMKSGKVLIMGGGAVGLAAIQLARAAGCSVAATAELVDYTSENTRQLTWRFDAVLDTIGVALGVNVLKRGGHYMTLQGEAVKLDAEGLEQIARLAKEGRLKIPVGFSLDEAAKAQTDSQKVVLQV>AT3G15090.1RDVSGEVAAIGTSVKSLKVGQEVFGTYTDYGILSELTEKPSSHVEASAIPFAALTAWRALKNARITEGRLLVFGGGAVGFSAIQLAVASGCHVTASAEQVDYTTEDIEAVKGKFDAVLDTIGGRIGINFLRKGGNYMTLQGEAASLDPEGLAEIQRLVGAGKLKIPVEFPITDVVAAHEQIPKVVLEF>BRADI_2g50080v3 isoform 1RDISGEVAATGTSVSSFFIGQEVFGTCADYAILSELTPKPSTHVEASAIPFAALTAWRALHTAGISEGRVLVIGGGAVGLSAVQLAVAAGCSVSATAEKIDYTSEDTEAVTGKFDAVLDTIGVRIGINLLRRGGHYMTLQGEAAALDPEGLHEIRRLSGAGKLQIPVEFPISQVREAHELVPKVVLEF>BRADI_2g50080v3 isoform 2--------------------------------------------EASAIPFAALTAWRALHTAGISEGRVLVIGGGAVGLSAVQLAVAAGCSVSATAEKIDYTSEDTEAVTGKFDAVLDTIGVRIGINLLRRGGHYMTLQGEAAALDPEGLHEIRRLSGAGKLQIPVEFPISQVREAHELVPKVVLEF>F56H1.6__RAD-8RDCTAVVESVGGDVHNLAPGDEVMGTHAEFVVTDYCSKKPSSFVSAAALPYVASTAYSAFTSQRNAKQRVLIHGGGGVGSMAIQLLKAWGCEIVATAIPVDYTSDQATELIEPFEVILDTVDSKWSDNVMGVNCVHVSIVSPLMRESSDLMSQLSRFAEDGKIVPVVEMGFEELEKAYENGRKTVIKY>NP_570962.2_RTN4IP1RDVSGVVMECGLDVKYFQPGDEVWGTLSEFVVVSEVSHKPKTHTQAASLPYVALTAWSAINSDRNCKGRALILGAGGVGTFAIQVMKAWGAHVTAVADEIDYTLGSVEQLKSLFDFILDNVGGTWALNFLKKGATYVTLVTPFLLNSGPYLDEIAELVDAGKIRPVIEFPFSEVPEAFLHARKTVVNV>NP_116119.2_RTN4IP1.1_RDVSGVVMECGLDVKYFKPGDEVWGTLSEFVVVSEVSHKPKTHTQAASLPYVALTAWSAINNDKNCTGRVLILGAGGVGTFAIQVMKAWDAHVTAVADDIDYKSGSVEQLKSPFDFILDNVGGTWAPDFLKKGATYVTLVTPFLLNSGPCLDDIAELVDAGKIRPVIEFPFSKVPEAFLHARKTVINV>NP_001305675.1_RTN4IP1.2_RDVSGVVMECGLDVKYFKPGDEVWGTLSEFVVVSEVSHKPKTHTQAASLPYVALTAWSAINNDKNCTGRVLILGAGGVGTFAIQVMKAWDAHVTAVADDIDYKSGSVEQLKSPFDFILDNVGGTWAPDFLKKGATYVTLVTPFLLNSGPCLDDIAELVDAGKIRPVIEFPFSKVPEAFLHARKTVINV>Mp6g05150.1YDCSGIVVKVGSEVSKFKVGDEVYGTLAQYTAAELLAKKPQTFAEAASLPLALQTAYQGWQKAGIKEGKVMVLGGGGVGTLAIQLAKVFKASVAATADVVDYTKQKLEEWPEKYDVVYDTVGKGEGTKVVKPDGGLVVITGPVEPPKGSDLEELNPYFETGSMQAVLDFPFTKVVEAFGRAFKVVISP>AT1G23740.1 Alkenal/one oxidoreductaseYDVAGVVVKVGSAVKDLKEGDEVYGSLAEYTAVELLALKPKDFAQAAGLPLAIETADEGLVRTEFSAGSILVLNGGGVGSLVIQLAKVYGASVAATADLIDYTKENIEDLPDKYDVVFDAIGMDKAVKVIKEGGKVVALTGAVTPPNGDVLKKLNPYIESGKVKPVVDFPFSRVADAFSHATKVVVYP>MA_10433694g0010 2-methylene-furan-3-one reductaseYDVAGIVIKVGSKVEGFKEGDEVYGSLGEYTAVELVALKPKDFTQAASLPLAILTAYEGLERAGFGPGSILVLGGGGVGSLVIQLARVFGASIAATTDIIDYTKQNFEELPEKYDVIYDAVGQDKAVKAIKGGGSVVALTGAVTPPNGSVLSKLNPYLESGEIKPIIDFSFSQLVEAFSRATKVVISP>XP_002971209.2_2-methylene-furan-3-one_reductase_YDVAGVVAKVGSEVSSLKQGDEVYGSIAQYTAVELLAIKPRSFIQAASLPLAIQTAQEGFDKAKLRAGSVLVLGGGGVGSLAIQLAKVYGASVAATADIIDYKEKNFEDMPERYDVVFDAVGEPKGTNVVKEGGTAVVLTGPVNPPNGSNLVRLKDFLESGTVKPVIDFDFGSVVDAFCRASKVVISV>XP_024543199.1_2-methylene-furan-3-one_reductase_YDVAGVVAKVGSEVSSLKQGDEVYGSIAQYTAVELLAIKPRSFIQAASLPLAIQTAQEGFDKAKLRAGSVLVLGGGGVGSLAIQLAKVYGASVAATADIIDYKEKNFEDMPERYDVVFDAVGEPKGTNVVKEGGTAVVLTGPVNPPNGSNLVRLKDFLESGTVKPVIDFDFGSVVDAFCRACKVVISV>Azfi_s0003.g007918YDVSGVIVKVGSEVTSFKEGDEVYGSIAQYTVTELLGIKPKSFAEAASLPLAIETALEGLERAGFKEGTLLVLGGGGVGSLAIQLAKVFGASVAATADVIDYKKEKFEDLPEKYDVVFDGVGEEKAVKVVKEGGSVIALTGPVTPPKGSSLAKLNPYLESGKVKPILDFSFSQVVEAFSRATKVVIAP>Azfi_s0123.g048404FDVAGVVVKVGSDVKKFKEGDEVYGTLAQYTVTEVLALKPKSFEEAASLPLALLTAQTGFEKAAFKPGSVLILGGGGVGTLAIQLAKVYGASVATTADVIDYTTTKYYELPEKYDFVYDTVGDANAAKAVKEGGAVLTIADSAKPPNGDNLAKLNPFLEEGKLKPVIDFPFSQVKEAFERARKLVIAP>MA_427213g0010 2-methylene-furan-3-one reductaseCDVAGVIVDEGDNVSKFSKGDEVYGTLTQYTAVELIASKPESFEEAASFPLALQTAQEGFDRVNFQRGSVFIVGGGGVGSLAIQLAKVYGASIATTADTIDYTKQSYDQIPQKFDFVFDTVGDHRSYVVAKEKAKIIDIANSPHPRSGSNLERLRGYIECEKLKPVIDYSFSNVMEAFKRARKIVISP>MA_207070g0010_2-methylene-furan-3-one reductaseCDVAGVIVDEGDNVSMFSKGDEVYGTLAQYTAVELIALKPESFEEAASFPLALQTAQEGFDRAKFLRGTVFIVGGGGVGSLAIQLAKVYGASIATTPDMIDYTKKSYDQILEKFDFVFDTIGDHRSYVVAKEEGKIIDIANSPHPRSGSNLERLRSYIECEKLKPVIDYAFSNVMEAFKRARKVVISP>MA_8441g0010_2-methylene-furan-3-one reductaseCDAAGVVVEVGDGVSKFKNGDEVYGTLAQYTVVELVAPKPTSFEEAASLPLALLTAQQAFDIAKFEKGSVFIVGGGGVGSLAIQLARVYGASIVSTADLIDYTKQSYDQISEKFDFVFDTIGDAKSHVVAKEEAKILDIASQPTSRHAKNLEKLQPYIESKKLKPVIDYSFSDVIEAFKRARKIVISP>MA_10436234g0010_2-methylene-furan-3-one reductaseCDAAGVVVDVGDGVSKFKNGDEVYGTLAQYTVVELVAPKPTSFEEAASLPLALLTAQQAFDIAKFEKGSVFIVGGGGVGSLAIQLARVYGASIVSTADLIDYTKQSYDQISEKFDFVFDTIGDAKSHVVAKEEAKILDIASQPTSRHAKNLEKLQPYIESKKLKPVIDYSFSDVIEAFKRARKIVISP>Mp1g20380.1 CEQORHTDIAGEVVKVGSEVKDFAPGDKVVGAMAQYAVANTTVVRPPSAVEAACLPVAALTALQALTFDGSYSGNVLVTGAGGVGTYAVQLGKIAGAHVTATADELDYKTPEGKKLISKYDLVVNAAASSDMQPQLAPKGVVFELTPSPKTFEARNLEMLVGFVKEGKLHAVIDFPLAKVEEAWKHAVKIVITV>AT4G13010 CEQORHTDVAGEVVEVGSGVKNFKAGDKVVGGLAEFAVATLTVKRPQGAAEAAALPVAGLTALQALTLDGTGKKNILVTAAGGVGHYAVQLAKLANAHVTATADELDYKTPEGAALKSKYDAVVHCANGSVFEPNLSENGKVIDITPGPNAMKAENLEFMVNLVKEGKVKTVIDHPLSKAEDAWAHATKIIVEP>BRADI_5g07080v3TDVAGVVVDVGPGVKDFIAGDQVVGGLAEYAVASLTVKRTPSAADGAGLPIAAGTALQALRFDGTGKPNVLITAAGGVGLYAVQLAKLANLHVTATGDEMDYKTPQGVSLQSKYDGVVHCTVGSVFKPLLSDTGRAIDITPNFSAINKADLEFLVGLLKDGKLKTVIDFPLSDASKAWQHATKIVVEM>Azfi_s0035.g025558TDIAGEVVSVGPGVNTFSVGDKVIGAFAEFAVASYTAKIPETAIDGCSLPVAAITALQAIRLDGTSKANLLITAAGGVGIYAVQIAKLTGAHVTATANELDYKTPEGDALKSKYDAIIHCATGSKFSSTLTPNGKVIDLTPNFKSINSEDLKLLGELVKDGKLRNIVDHPFAKAEESWVHATKVVVTF>Azfi_s0035.g025557TDIAGEVVSVGPGVKTFSVGDKIMGSLAEFAIAIFTVKRPESAIDGCSLPVAGMSALQAIRLDGASKANLLITAAGGVGTYALQIAKLTGAHVTGTADELDYKTPEGEALKSKYDAIIHCATDSKFSSNLSPNGKVIDLTPNFKSFNAEDLKLLGDLVKDGKLRTIVDYPLEKAEAAWVHATKVVVNH>Azfi_s0002.g001541TDVAGEVVAVGPGVDAFTTGDKVLGSLAEFAVVPSTVKRPLSAVDACCIPVAGLTALQAIKLDGKSDENLLITAAGGVGLYAVQIAKLTGAHVTATADELDYKTPEGAALQSKYDAVIHCARGSTFSPVLKPAAKVVDLTPDLKSLKAEDLEVLVALARDGKLKTIVDYPLARSGEAWAHSTKVVVEV>XP_002975582.2_CEQORHTDVSGEVVSVGPGVTGFSQGDKVIGGFAQYAVAGYTAKRPSSAVEAAALPVAGLTAYQSLRYDGSYKGNVLITAAGGVGTYAVQLAKLAGAHVTATADELDYKTPEGAALKSKYDVVLQCATTSTFQPVLSSKGKVIDLTPSFGVMNAKDLGELAELVNEKKVKSLIDFPLDKAEDAWAHATKIVVTN>MA_959201g0010_CEQORHVDVAGEVVSVGPGVNDLTTGDKIVGGLAEYVVAPTTVKRPSSAENGAGLPTAALSALQAIRLDGSGKENLLITAAGGVGLYAVQIAKLGRAHVTATADELDYKTPEGAALKSKYDAVIHCTSNSTFQPNLSPNGKVIDLTPNFMSINSKDLALIVRLVNEGKVKTVIDYPLEKAADAWAHATKIVVGK>BRADI_4g34400v3CELAGEVVELGSGVSGFAAGDKVIGGLAEYAVVSCAATRPPSATEGACLPIAAATALAALRLDARGPPNVLVTAAGGVGTFAVQLAKLSGKHVTVTANDLDYGTPEGAALRGKHDAVVHCAEGSAFEPALADGGVVVDLTARVASVKKEDMEVLLGLVRRGQIRVVIDYPLSRAHEGWAHATKIVVEM>BRADI_5g06207v3SDVAGEIVEVGAAVSEFKVGDRVVGGLAEYVAASVTVALPASTVDAAGLPVAGLTALQAVRFDGTGSGNILITAGGGIGTYAVQLAKLGNHNVTATADELDYKTPEGAALKNKYDYIVNTTNASAFKPALSSHGRVVDLAPNLANFGMEDLRFLLELVKEGKLKTVVDHPFDKAADAWEHATKVIVEM>XP_002970156.2_CEQORHSDISGEVVSLGPGVTGFSVGDKVVGGLAKYASAEFTTRIPSSPIEAAGLPIAGLTALYSLQIPSKDFQTILVTAAGGVGTYAVQLAKLTGAHVVATADELDYKTPQGAKLQTKFDVIIHCAYHSTFEHVLTRKGIVVDITPASESTHTSGIEALVDMMQKGKLKTIIDHPISNVTAAWFHATKIVVTL>XP_002978323.2_CEQORHSDISGEVVSLGPGVTGFSVGDKVVGGLAKYASAEFTTRIPSSPIEAAGLPIAGLTALYSLQIPSKDFQTILVTAAGGVGTYAVQLAKLTGAHVVATADELDYKTPQGAKLQSKFDVIIHCAYHSTFEHVLTRKGIVVDITPASESTHTSGIEALVDMMQKGKLKTIIDHPISNVTAAWFHATKIVVTL>Mp1g05710.1LECSGVVEAVGDRVQRWKVGDEVCGGYAEKINVPQVLPVPKSLLHAAALPEVSCTVWSTVFTSKLTAGSFLVHGGSGIGTFAIQIAKAKGAKFCTVADVINYKEQDFVVVKEGVNVILDHIGAQKNVDALSVDGRLFIIGFMGGAAVAEVLKHVWPEVESGKVKPVVHFPLGDACKGHEAHVKIILTA>Azfi_s0211.g058133LECSGTIESVADDVQHWKVGDQVCGGYAEKVSLPHLLPIPKSLLDAAGFPEVACTVWSTVFTSHLSGGSFLVHGGSGIGTFAIQMAKYMGVRFCTVADVINYKEDDFVRVKEGVNVILDNIGAERNLDALSVDGRLFIIGFQGGAKVQEIRTHVWPAIESGKVKPVIYFPLAEAPDAHRRHIKILLTT>XP_002960362.1_quinone_oxidoreductase_PIG3_LECSGVIERLGARVDGWKVGDEVCGGYAEKVAVAQLLPIPRSLRDAASLPEVACTVWSTVFACHLSAGSVLIHGGSGIGTFAIQMAKSIGAQLATAAEVINYKDDDFVRVKEGVDVILDMVGAERNLEALGMDGRLFIIGFQGGASVAEVWQNVWPAVEEGKVKPVIYFPLEEAAQALQQHFKILLTP>XP_002967353.1_quinone_oxidoreductase_PIG3_LECSGVIERLGARVDGWKVGDEVCGGYAEKVAVAQLLPIPRSLRDAASLPEVACTVWSTVFACHLSAGSVLIHGGSGIGTFAIQMAKSIGAQFATAAEVINYKEDDFVRVKEGVDVVLDMVGAERNLEALGMDGRLFIIGFQGGASVAEVWQNVWPAVEERKVKPVIYFPLEEAAQALQQHFKILLTP>AT4G21580.1LECSGTIESVGKGVSRWKVGDQVCGGYAEKVSVPQIFPIPASLKDAAAFPEVACTVWSTVFMGRLSVGSFLIHGGSGIGTFAIQIAKHLGVRFVTAADVINYKTEDFVKVKAGVDVILDCIGAQKNLDSLNFDGRLCIIGLMGGANVREVEKNVWPAIEAGKVKPVIYLPLSQAAEGHSNHIKILLET>Azfi_s0002.g004335LEVAGQVVALGDGAAGFAVGDKVCGAYAEYCAVPQALAWPQDAIRAAALPETFFTVWANLFMAGLKEGSVLIHGGSGIGTTAIQLAQALGATFVTVAARINYKSEDFAVVKRGVDVILDMIGADRNLACLARDGRLSIIAFLGGAVRDQLQAKVWPLLEAGTVAPVINIKGRQAATCACDAVKIVVDV>F39B2.3KDGAGFVELVGESVKNVKVGDRVWDSTAEYVAVNRPFELPESFEEGASLGVPYLTAYRALFLAGAKTGVILVHGAGGVGSALMQLAAWRNIEVGTAARNYNHSDKQYVKMKNGFNHIFEMAAHNTDLGLLAPRGRVAVIGNRAETTLLDFGINIVSFLKETEFRPLINYRLEQLGLAHEKGANLVVQI>NP_001344601.1_quinone_oxidoreductase_SDVAGIIESVGDKVSAFKKGDRVFGGYAEFALAATIYPLPENFRQGAALGIPYFTACRALFSARARAGSVLVHGAGGVGLATCQIARAHGLKLGTAAHEFNHKEANYIKIKMGVDVIIEMLANSNDLKLLSHGGRVVVVGCRGPIEFQQFAGLLQAGIEKGWVKPVIGYPLEKAAQAHESGKKMILLL>NP_001123514.1_quinone_oxidoreductase_a_SDVAGVIEAVGDNASAFKKGDRVFGGYAEYALAATVYKLPEDFKQGAAIGIPYFTAYRALISACVKAGSVLVHGAGGVGLAACQIARAYGLKLGTAAHEFNHREVNYIKIKKGIDIIIEMLANSKDLSLLSHGGRVIVVGSRGTIEFQQYAAALQAGMEIGWLKPVIGYPLEKVAEAHESGAKMILLL>NP_001123515.1_quinone_oxidoreductase_b_SDVAGVIEAVGDNASAFKKGDRVFGGYAEYALAATVYKLPEDFKQGAAIGIPYFTAYRALISACVKAGSVLVHGAGGVGLAACQIARAYGLKLGTAAHEFNHREVNYIKIK--------------------------VVGSRGTIEFQQYAAALQAGMEIGWLKPVIGYPLEKVAEAHESGAKMILLL>Mp3g08280.1 NADPH_quinone reductaseGDFSGVVTEAGAKVTHVKVGDRVCGSFAEEFVADEMFLIPSDLVAAGALPVAFGTSHIALDRANLRPGVLLVLGAGGVGLAAVELGKLMGAIIAVAADFLDSSEDGIIPVQAGVDVLYDPVGGKEALKLVKWGGQILVIGFASGEILRDSMKQLLRWAAEGKLDVHISFPLSQANLAFAKAIKVIFTL>AT3G56460.1SDYSGIVDAIGPAVTKFRVGDRVCGSFAQFIVADRLFLVPEDMVAAAALPVAFGTSHVALVRARLTSGVLLVLGAGGVGLAAVQIGKVCGAIIAVAVDHVDLGTENVISVKEGVDVLYDPVGGKESMKVLKWGAQILVIGFASGEILEDSIKELLSWLSRGLITIHISYSLSQANLAFGKAIKVMIAL>NP_009602.1_NADPH_quinone_reductaseREASGTVVAKGKGVTNFEVGDQVASTFAQYSKISPVMKLPKSDEEYAAGLLQVLTALSFTNAYHVKKGYVLLFAAGGVGLILNQLLKMKGAHIAVAAEYINASKEDILQVLKGVDASFDSVGKEISLAALKRKGVFVSFGNASGLIWKYYSDEFFGLVNSKKLNIKIYYPLRDYRTAAAKTVKLVLEI>Mp7g12910.1FEAVGVVASLGEGVDSLVPGSPVAGGFSEFCQVPNVIPMPVAVPELVALLTSGLTASLALEAGRIKSGTVLVTAAGGTGQFAVQLAKLAGNTIATCVDRIDYKKESIKVLKKGIDLIYESVGGTTCLNALARRGRLIVIGMISQYQWRDHAARLTKLYLDKKLKVTVDAGVEAIADAVEQSLKVVVRL>AT1G49670.2FEGVGLIAAVGESVKNLEVGTPAAGAYSEYMIVSHVLPVPRPDPEVVAMLTSGLTALIALEAGQMKSGTVLVTAAGGTGQFAVQLAKLSGNKIATCVDRIDYKSENIKVLKKGVNIIYESVGGDMCLNALAVYGRLIVIGMISQYQWKQNLDKLFNLYALGKLKVGIDIGLNAVADAVEKSTKVVVCI>NP_666202.2_prostaglandin_reductase 3FEGIGEVVALGLSASAYTVGQAVAGSFAEYTVVPIAIPMPSVKPEYLTMLVSGTTAYLSLELGELSEGKVLVTAAGGTGQFAVQLSKIAKCHIGTCCDRINYRTEPVEVLKQGVDVVYESVGGDLAVDALATKGRLIVIGFISGYQYQAAMERLLELYARGDLVCEVDIGLESVFQAVDKNTKLVVEL>NP_787103.1_prostaglandin_reductase_3.1_FEGIGEVVALGLSASAYTVGQAVAGSFAEYTVVPIATPVPSVKPEYLTLLVSGTTAYISLKLGGLSEGKVLVTAAGGTGQFAMQLSKKAKCHIGTCCDRINYKTEPVGVLKQGVDVVYESVGGDLAVDALATKGRLIVIGFISGYQYQAAMSHLLEMCVSGDLVCEVDTGLESIFRAVNKNTKIVVEL>NP_001293022.1_prostaglandin_reductase_3.2_------------------------GSFAEYTVVPIATPVPSVKPEYLTLLVSGTTAYISLKLGGLSEGKVLVTAAGGTGQFAMQLSKKAKCHIGTCCDRINYKTEPVGVLKQGVDVVYESVGGDLAVDALATKGRLIVIGFISGYQYQAAMSHLLEMCVSGDLVCEVDTGLESIFRAVNKNTKIVVEL>R04B5.5HETSGIVSEVGNEVKHLKVGDRIAGTLSRFVVHDFCFKLPDSFEDGALIE-PLSVAIHACRRGNVQMGRVLVLGAGPIGVLNLITAKAVGAKVITDADAINVKGKSVKEIITQPDVCIECTGAETAITTTKSGGVIVLVGLGADRVYVNCYPTAIELISSGKLNLSGLYKLEETQEAFKDVIKVFIQC>R04B5.6HESSGVVSEIGSEVKGFKVGDRIAGALSRFVVHDFCFKLPDSFEDGALLE-PLSVAIQACRRGTVQMGKILVLGAGPIGVLNLLTAKAIGAKVITDADAINVMGKRVREIIKQPHVSIECTGVETAIMTTRSGGVVVLVGLGAERVSANCYSTAIELISSGKLDLSGLYKLEESLEAFKDVIKVFIHC>D2063.1HEGAGSVISVGSKVKNWQIGDKVGGTFQEYATIRDAIKIPKNMAAAAPVLCGGVTAYKALKESEVKSGIVAVTGAGGLGSFAIQYARAMGMRVAI-AEWVDAFGTEIVHIREAHGVVNFAAAKEKALEYVRKRGTVVFVGLAKDSKSRLDVNEAMDFVARGAVNVPLEVKLEDVAEVYTKINRVVVDF>NP_009703.3_ADH5_HEGAGVVVKLGSNVKGWKVGDFAGGTFQEYATADQAAHIPPNLAEVAPILCAGITVYKALKRANVIPGWVTISGAGGLGSLAIQYALAMGYRIGIDGEIIDFTEEKIVAIIKSHGVINVSVSEEASTRYCRPNGTVVLVGMPAHAYNRADTREALDFFARGLIKSPIHAGLSDVPEIFAEIVRYVVET>NP_014555.1_ADH1_HEGAGVVVGMGENVKGWKIGDYAGGSFQQYATADQAAHIPQDLAQVAPILCAGITVYKALKSANLMAGWVAISGAGGLGSLAVQYAKAMGYRLGIDGEVIDFTKEKIVAVLKAHGVINVSVSEEASTRYVRANGTTVLVGMPAGAKNRADTREALDFFARGLVKSPIKVGLSTLPEIYEQIVRYVVDT>Mp1g09240.1HEFMGVVHEVGPDVKSVKKGDRVVGGQAEYVRVPNTLKVPESDEKVVLLSDILPTAWHANELGEVGQGVVAIWGAGPVGILAAHCAQARGAEFLIDGLRINFKQEKVYALRKGIDVGIDAVGMDSVLHKLQMGGRVSIVGAYAGYTVQKYWKHLLELVQKSQLNPSLVQPLEKAPELYKVDNKVVMHP>AT3G45770.1YEGVGEVYAVGSNVNGFSPGDWVIGTWQTYVVKEVWHKIDKPMEYAATITVNPLTALRMLEFVNLNSGSVVQNGASIVGQCVIQLARLRGISINLAADEFSESQLNVKNVKSEPALGFNCVGGSLVLKYLREGGTMVTYGGMSKKPCREMIDYLLGLARDGKLKYETEVPFEEFPVALDKALKLVITF>NP_013872.1_Yim1p_RDYSGVIIKAGKDVDNWKVGDKVNGTLTHYLILNHMVEVPKNDPYAAAWPLTFGTAFSTLYKKDWTSDKVLVIGATSVSYAFVHIAKYFNIGVVGIYDYVPYDEGSIVNVKKKFDMIFDSVGNPVIDQFLKPKAKYVTIAGNNKADPNNFIEVGNEMIKKGTYKPPIDYEFDQYKEAIDRAKKVVVKM>NP_009484.2_Ast1p_REYSGVITEVGENLNYWHVGDEVYGCLQSSILVDPILLRPESAEEAAGSLFCLATGYNILNNKYLKQDNVLINGGSSVGMFVIQLLKHYKLKLVIVDEMIDYLTCRSSPLRKKFDVVLDFVGGSHSSSLIHGGGAVTTVGDVANYKNNDWIEQCGDFLKNGTVKCVVDYDWKDHKEAFSRAQKLIMNV>NP_598440.1_quinone_oxidoreductase-like 1_REVSGIVLEVGRKVTFFQPDDEVVPGLCEVIRVHYLVHKPESWTEAAGVIRDGVRACTALYLSQLSPGSVLIMDGSAFGTIAIQLAHHRGAKISTAIARIDVSNGKVASCLEGVDIVIDAGVRKDDEPAVKLKHDLLGVGGHWVTTYLCILKDVMEKLSAGVFRPLLDIPLYEAKVSMEQERKQVVQF>NP_665857.2 quinone oxidoreductase-like 1REIAGIVLDVGSKVSFFQPDDEVVPGLCEVVRVHYLVHKPETWTEAAGSIRDGVRAYTALHLSHLSPGSVLIMDGSAFGTIAIQLAHHRGAKISTAIARIDVSNGKVASCLEGVDIVLDAGVRKDDEPAVKLKHDLLGVGGHWVTTYLCILKDVMEKLSTGVFRPQLDIPLYEAKVSMEQGRKQVVQF##Fig3C_tree##(NP_598440.1 quinone oxidoreductase-like 1:0.06249885,NP_665857.2 quinone oxidoreductase-like 1:0.02259909,((((Mp1g09240.1:0.85839406,(R04B5.5:0.05781637,R04B5.6:0.12672612)0.999800:0.79211118)0.909600:0.33188661,((F39B2.3:0.69261333,(NP_001344601.1 quinone oxidoreductase:0.13893507,(NP_001123515.1 quinone oxidoreductase b:0.00000001,NP_001123514.1 quinone oxidoreductase a:0.00000001)0.904600:0.08140509)0.993600:0.36346995)0.990000:0.35282640,(Azfi_s0002.g004335:0.53406613,((XP_002967353.1 quinone oxidoreductase PIG3:0.01074394,XP_002960362.1 quinone oxidoreductase PIG3:0.01101734)0.985500:0.16573244,(Mp1g05710.1:0.20030641,(Azfi_s0211.g058133:0.11185949,AT4G21580.1:0.23637114)0.242300:0.06222664)0.826200:0.07567756)0.985600:0.24402746)0.987100:0.32158862)0.946500:0.19719576)0.376800:0.05515729,((NP_009602.1 NADPH:quinone reductase:0.95385763,(((NP_666202.2 prostaglandin reductase 3:0.00000001,(NP_787103.1 prostaglandin reductase 3.1:0.00000001,NP_001293022.1 prostaglandin reductase 3.2:0.00000001)0.998000:0.12164428)1.000000:0.50750967,(Mp7g12910.1:0.28674816,AT1G49670.2:0.19753166)0.832700:0.08902724)0.999900:0.47165463,(AT3G45770.1:1.11648933,(D2063.1:0.34340223,(NP_009703.3 ADH5:0.13811770,NP_014555.1 ADH1:0.08780730)0.998400:0.40191363)0.999500:0.58425156)0.163800:0.06109361)0.718500:0.08216700)0.656200:0.05891577,(Mp3g08280.1 NADPH_quinone reductase:0.23062824,AT3G56460.1:0.24322269)1.000000:0.56644032)0.765300:0.07456405)0.919700:0.18241052,((Ast1p:0.76128830,Yim1p:0.90448948)0.968600:0.31518750,((Azfi_s0123.g048404:0.09733407,(((MA_10436234g0010 2-methylene-furan-3-one reductase:0.00000001,MA_8441g0010 2-methylene-furan-3-one reductase:0.00521802)0.994800:0.14895738,(MA_207070g0010 2-methylene-furan-3-one reductase:0.04997770,MA_427213g0010 2-methylene-furan-3-one reductase:0.04946635)0.965100:0.10555962)0.999900:0.27230476,(Mp6g05150.1:0.31028860,((XP_024543199.1 2-methylene-furan-3-one reductase:0.00532293,XP_002971209.2 2-methylene-furan-3-one reductase:0.00000001)1.000000:0.22780587,(Azfi_s0003.g007918:0.12852051,(MA_10433694g0010 2-methylene-furan-3-one reductase:0.14710018,AT1G23740.1 Alkenal/one oxidoreductase:0.18059765)0.954100:0.07858614)0.365800:0.04039020)0.879100:0.07016225)0.962600:0.11049078)0.743600:0.08185893)1.000000:0.38930292,((((AT3G15090.1:0.12462564,(BRADI_2g50080v3 isoform 2:0.00000001,BRADI_2g50080v3 isoform 1:0.00000001)0.998400:0.16755920)0.987200:0.19913568,(Mp3g19030.1 MpRTN4IP1L:0.14767877,P_024530385.1 RTN4IP1 homolog:0.22758607)0.746500:0.06465071)0.999700:0.42159450,(Mp1g20380.1 CEQORH:0.26113432,((XP_002970156.2 CEQORH:0.00515367,XP_002978323.2 CEQORH:0.00000001)1.000000:0.45612014,(XP_002975582.2 CEQORH:0.17747816,(((BRADI_4g34400v3:0.40783459,BRADI_5g07080v3:0.22021143)0.854000:0.06436813,(BRADI_5g06207v3:0.34018703,AT4G13010 CEQORH:0.16764354)0.051700:0.02719531)0.860700:0.06880628,(MA_959201g0010 CEQORH:0.19798491,(Azfi_s0002.g001541:0.22169149,(Azfi_s0035.g025557:0.10726838,Azfi_s0035.g025558:0.09947070)0.980600:0.10905203)0.889500:0.08412675)0.818400:0.05644286)0.936800:0.07850020)0.105200:0.03650927)0.580100:0.09077161)1.000000:0.54629892)0.452100:0.10691551,(F56H1.6 RAD-8:0.68067663,(NP_570962.2 RTN4IP1:0.02915728,(NP_001305675.1 RTN4IP1.2:0.00000001,NP_116119.2 RTN4IP1.1:0.00000001)0.965900:0.06077562)0.990700:0.27510796)0.969100:0.22457996)0.915100:0.14543136)0.000000:0.03238224)0.870600:0.15015214)1.000000:1.29727537);
